# Enhancing Interfacial Charge Transport in Gold Nanoparticle@Polyaniline Hybrids via *N*‐Heterocyclic Carbene Linkers

**DOI:** 10.1002/anie.202526136

**Published:** 2026-05-11

**Authors:** Ningwei Sun, Haoran Zhang, Ziwei Zhou, Po Yuen Ho, Ilka Hermes, Yanfei Gao, Shivam Singh, Dmitry A. Ryndyk, Olga Guskova, Zhenyang Jia, Tathagata Chatterjee, Antoine E. Jimenez, Kaline Pagnan Furlan, Marina Sebastian, Christian Rossner, Stephan Link, Christy F. Landes, Yana Vaynzof, Andreas Fery, Franziska S.‐C. Lissel

**Affiliations:** ^1^ Leibniz Institute of Polymer Research Dresden Dresden Germany; ^2^ Hamburg University of Technology Hamburg Germany; ^3^ Chair For Emerging Electronic Technologies TUD Dresden University of Technology Dresden Germany; ^4^ Leibniz Institute For Solid State and Materials Research Dresden Dresden Germany; ^5^ Chair For Theoretical Chemistry TUD Dresden University of Technology Dresden Germany; ^6^ Department of Chemical and Biomolecular Engineering University of Illinois Urbana‐Champaign Urbana Illinois USA; ^7^ Department of Chemistry University of Illinois Urbana‐Champaign Urbana Illinois USA; ^8^ Karlsruhe Institute of Technology (KIT) Institute for Applied Materials (IAM) Ceramic Materials and Technologies Karlsruhe Germany; ^9^ University of Chemistry and Technology Prague Prague Czech Republic; ^10^ Department of Electrical and Computer Engineering University of Illinois Urbana‐Champaign Urbana Illinois USA; ^11^ Materials Research Laboratory, University of Illinois Urbana‐Champaign Urbana Illinois USA; ^12^ Chair For Physical Chemistry of Polymeric Materials TUD Dresden University of Technology Dresden Germany

**Keywords:** charge transport, electrochemistry, gold nanoparticles, interface, n‐heterocyclic carbenes

## Abstract

*N*‐Heterocyclic carbenes (NHCs) have emerged as a unique class of ligands for gold nanoparticles (Au NPs), combining strong metal binding with intrinsic electronic conductivity. Yet over the past decade, studies on Au NP@NHC systems have primarily focused on their stability, while the conductivity of NHCs has remained largely unexplored due to synthesis challenges. Here, we present a synthetic strategy that addresses this gap by employing amino‐functionalized NHC‐Au complexes with in situ oxidative polymerization of polyaniline (PANI) to yield electronically coupled Au NP@NHC‐PANI hybrids in aqueous media. This strategy enables both a controlled PANI shell growth and introduction of an electronically active NHC interlayer. Single‐particle scattering spectroscopy reveals that NHCs improve the interfacial electronic coupling as evidenced by pronounced plasmonic linewidth broadening. Conductivity measurements further confirm that NHCs enhance charge transport: conductive atomic force microscopy (C‐AFM) shows an increase in contact current from 14.6 to 99.4 pA under a 300‐mV bias, while lateral four‐probe conductance increases from 0.17 to 3.5 nS. These results provide the first direct experimental evidence of the conductive role of NHCs in hybrid NP‐polymer systems, establishing a new interface‐engineering strategy for the rational design of electronically delocalized nanostructures and their applications in nanoelectronics.

## Graphical abstract

An amino‐functionalized NHC‐Au complex is employed as a conductive molecular linker to couple large Au NPs with PANI, yielding Au NP@NHC‐PANI hybrids with an electronically well‐defined interface. SAMs of these hybrids reveal enhanced charge transport compared with Au NP@PANI. This work provides the first experimental evidence that NHCs can improve interfacial charge transport in Au NP‐CP systems, establishing a new strategy for engineering high‐performance hybrid electronic materials.

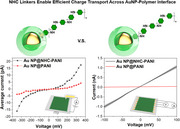

## 1 Introduction

Hybrid organic–inorganic materials integrate the advantages of both components, making them highly valuable in advanced material design [1, 2, 3, 4, 5]. Among them, hybrids of gold nanoparticles (Au NPs) and conducting polymers (CPs) are particularly attractive for applications in printed and flexible electrodes since they can combine the high conductivity of Au NPs with the processability and mechanical flexibility of CPs [6, 7, 8, 9, 10, 11]. Such composites enable direct fabrication of conductive patterns without the need for post‐sintering, which is a key advantage for cost‐efficient and scalable printed electronics [12].

While prior efforts have focused on improving the intrinsic conductivity of the individual components via polymer doping, nanoparticle assembly or post‐processing treatments [13, 14, 15], the critical role of the Au NP‐polymer interface has remained comparatively underexplored. Realizing the efficient charge transport across this interface is essential to fully unlock the potential of these hybrid systems, particularly in applications where performance is limited by interfacial barriers.

*N*‐heterocyclic carbenes (NHCs) offer a promising yet underutilized strategy for interfacial engineering in hybrid organic–inorganic nanomaterials. Compared with conventional thiol ligands, NHCs form stronger and more stable covalent bonds with gold surfaces, providing the Au NPs with exceptional thermal and electrochemical stability [16, 17, 18, 19, 20, 21, 22, 23, 24, 25, 26, 27, 28, 29, 30, 31]. Moreover, their delocalized π‐systems and conductive Au─C bonds can facilitate electronic coupling between the NP core and the surrounding CP ligands, making NHCs well‐suited as molecular linkers in charge‐transporting nanocomposites [32, 33, 34].

Despite these advantages, the synthesis with incorporation of NHCs into Au NP‐CP hybrids is challenging, especially for larger Au NPs (>20 nm). Existing approaches to Au NP@NHC rely either on (i) bottom‐up synthesis via the reduction of NHC‐Au complexes [23, 24, 28, 30], typically yielding ultrasmall particles (<7 nm), or (ii) a top–down ligand exchange, where NHCs are introduced onto preformed Au NPs [25, 29, 35, 36]. In the latter case, NHCs can be transferred as free carbenes, CO_2_ adducts or gold complexes. However, the ligand exchange using free carbenes in aqueous media is hindered by their instability. Besides, the direct synthesis of CP‐functionalized NHC‐Au complexes or NHC‐CO_2_ adducts has rarely been reported, likely due to the complexity of the multistep synthesis. As a result, there is still a lack of experimental evidence demonstrating whether NHCs can enhance charge transport across Au NP‐CP interfaces.

To address these challenges, we present a novel strategy by employing an amino‐functionalized NHC‐Au complex as a molecular linker between Au NPs (>50 nm) and polyaniline (PANI). This design not only enables efficient ligand exchange on Au NPs but also provides reactive amino groups that initiate in situ oxidative polymerization of PANI directly from the Au NP surface. Successful NHC functionalization and controlled PANI growth were confirmed, yielding Au NP@NHC‐PANI hybrids that can be assembled into well‐ordered monolayers for systematic conductivity studies. Comparative conductivity measurements with Au NP@PANI reveal a pronounced enhancement in interfacial charge transport, for the first time, providing the direct experimental evidence of NHCs in improving interfacial charge transport. Overall, this work establishes an interface engineering strategy for tailoring charge transport in hybrid nanostructures and opens new opportunities for high‐performance printable electronics.

## 2 Results and Discussion

**FIGURE 1:**
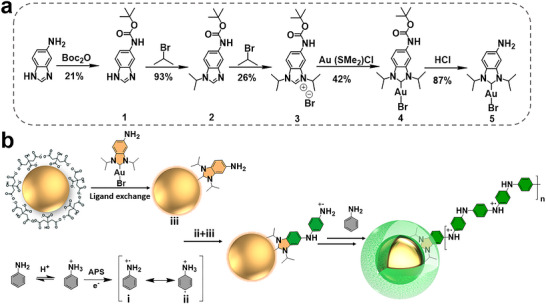
(a) Synthetic route to the amino‐functionalized NHC‐Au complex (**5**). (b) Schematic illustration of the ligand exchange of amino‐NHC onto Au NPs and the subsequent polymerization of aniline to form Au NP@NHC‐PANI composites.

To develop electronically coupled hybrid nanocomposites, an amino‐containing NHC‐Au anchor was first designed and synthesized (Figure 1a). The 1,3‐diisopropyl‐benzimidazolylidene framework was selected based on established structure‐binding correlations of benzimidazole‐derived NHCs on gold surfaces [37]. These correlations indicate that intermediate steric bulk at the wing‐tip positions favors upright, adatom‐mediated binding geometries while maintaining strong Au─C bonding. Such an upright orientation is advantageous in the present system as it minimizes steric‐induced tilting and promotes a well‐defined electronic interface between the Au NP core and the PANI shell.

The primary amine group of 5‐aminobenzimidazole (**1**) was protected with a *tert*‐butyloxycarbonyl (Boc) group to prevent side reactions in subsequent synthetic steps. Alkylation of **1** with 2‐bromopropane yielded 5‐Boc‐amino‐benzimidazole (**2**), which was then converted to 5‐Boc‐amino‐1,3‐diisopropyl‐benzimidazolium bromide (**3**) through a second alkylation with another bromopropane under mild conditions, avoiding side reactions with the amide. The synthesis of (5‐Boc‐amino‐1,3‐diisopropyl‐benzimidazolium) gold bromide (**4**) was achieved by reacting compound **3** with Au (SMe_2_)Cl. Subsequent Boc deprotection under acidic conditions yielded the target compound, (5‐amino‐1,3‐diisopropyl‐benzimidazolium) gold bromide (**5**). The successful synthesis of these compounds was confirmed by using nuclear magnetic resonance spectroscopy (NMR) and Fourier‐transform infrared spectroscopy (FTIR) techniques (Figures S4–S12).

With the amino‐NHC‐Au complex in hand, ligand exchange was performed according to a reported protocol [25, 38]. The ligand exchange is proposed to proceed via a transmetalation‐to‐surface mechanism, in which the NHC ligand chemisorbs onto the Au(0) surface to form a strong Au─C (NHC) bond, thereby replacing the original surface ligands (citrate or CTAC). To optimize the ligand exchange conditions, **5** (1 mM in acetonitrile) was added into 2.5 mL Au NPs aqueous solution by increasing the volume from 0.5 to 3 µL. At additions beyond 2 µL per 2.5 mL solution, NP aggregation was observed, indicating excessive ligand exchange and loss of colloidal stability. Optimal conditions were found to be below 1 µL, yielding stable Au NPs bearing mixed surface ligands (NHC together with residual original ligands).

Based on the amount of NHC ligands added, a rough estimate indicates that approximately 1,895 NHC ligands could be bound to the surface of each Au NP, corresponding to 0.24 molecules per nm^2^. The surface grafting density was further quantified using a fluorescamine assay [39, 40] (Figures S1 and S2). In this approach, the concentration of free (non‐surface‐bound) NHC remaining in solution after grafting was determined following nanoparticle removal by centrifugation. By varying the target NHC feed concentration, a Langmuir‐type adsorption isotherm was constructed (Figure S3). From this analysis, a saturation surface coverage of 4.0 NHC molecules per nm^2^ and a free enthalpy of adsorption of ‐56 kJ/mol were obtained. The determined saturation grafting density is consistent with previous results [41, 42, 43] on the formation of NHC monolayers on gold. The binding free enthalpy, which contains contributions also from the replacement of initial citrate ligands during grafting and entropic contributions, is larger than the values obtained for dithioesters and trithiocarbonates (‐36 kJ/mol) [44, 45]. According to the Langmuir model, at the experimental feed corresponding to a nominal grafting density of 0.24 molecules per nm^2^, the actual grafting density is predicted to be essentially identical (within two significant digits), indicating near‐quantitative surface binding under the optimized conditions.

**FIGURE 2:**
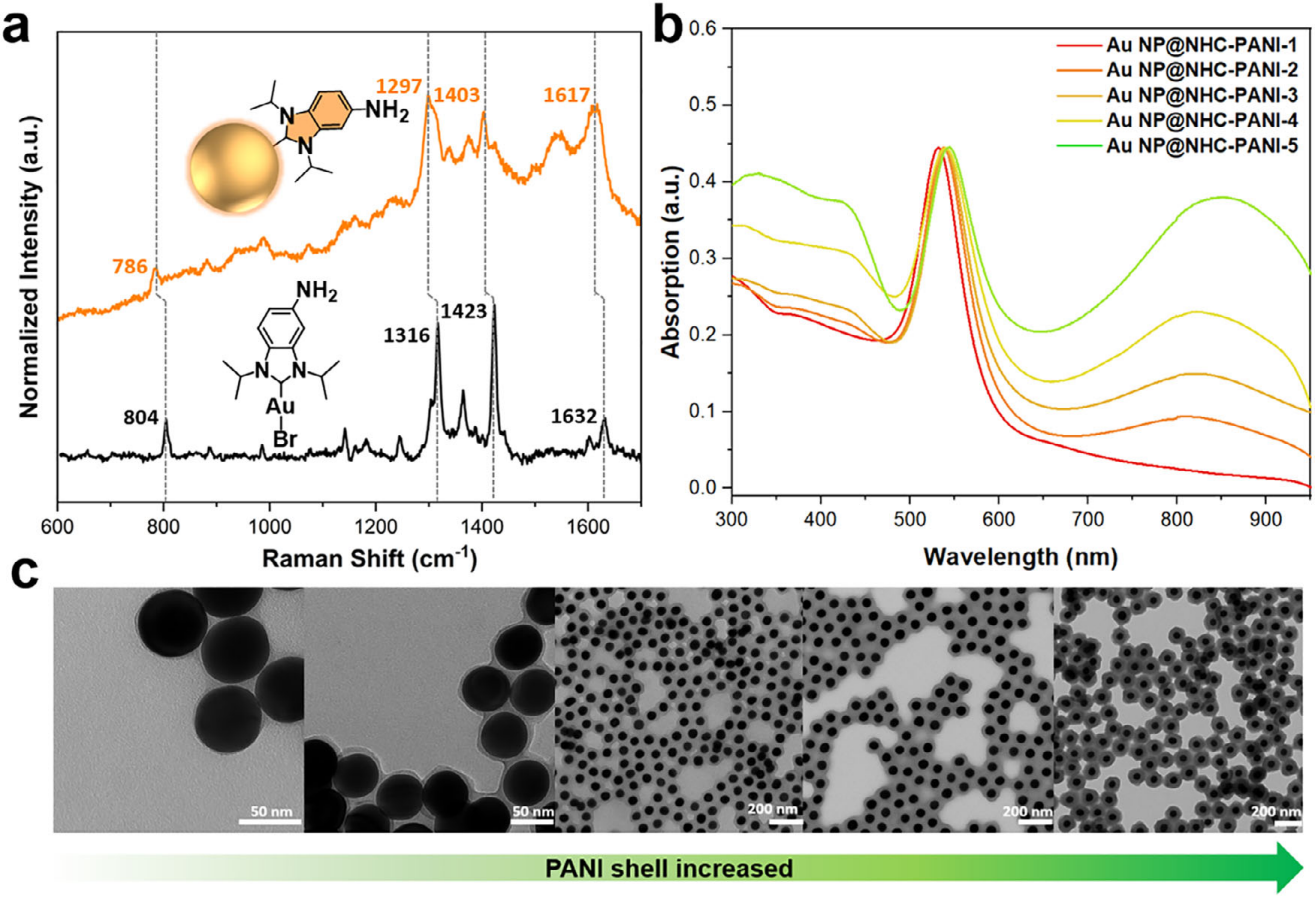
(a) Raman spectra of the amino‐NHC‐Au complex (**5**) and Au NPs after ligand exchange with amino‐NHC. (b) UV‐vis absorption spectra of Au NP@NHC‐PANI composites with increasing PANI shell thickness. (c) TEM images of Au NP@NHC‐PANIs with different PANI shell thicknesses.

The successful ligand exchange was also verified by Raman spectroscopy. After thorough washing to remove any residual, unbound ligands, the Raman spectra of **5** and the Au NPs after ligand exchange were comparatively analyzed. The Raman spectrum of **5** (Figure 2a) exhibited characteristic bands at 804, 1316, 1423, and 1632 cm^−1^. The band at 804 cm^−1^ can be assigned to a mixed vibrational mode involving Au‐C stretching coupled with aromatic ring in‐plane deformation and the wing‐tip mode. The bands at 1316 and 1423 cm^−1^ arise from coupled Au‐C stretching and wing‐tip vibrations, while the band at 1632 cm^−1^ corresponds to aromatic ring in‐plane stretching [46]. After ligand exchange, these characteristic vibrational features are retained in the spectrum of the functionalized Au NPs, confirming the presence of the NHC backbone on the NP surface. Notably, systematic red shifts of approximately 15–20 cm^−1^ are observed for these bands. Such shifts are consistent with previously reported SERS spectra of NHC‐Au systems, where coordination to anchoring onto Au NPs leads to a decrease in vibrational frequency due to electronic interaction and surface‐induced mode softening. These spectral changes provide strong evidence for successful anchoring of the amino‐functionalized NHC‐Au complex onto the Au NP surface, thereby confirming the effectiveness of the ligand exchange process.

Having established a robust NHC anchoring strategy, PANI was grown directly from the amino‐functionalized Au NP surfaces via surfactant‐assisted oxidative polymerization (Figure 1b). Detailed procedures for the polymerization and characterization are provided in the Supporting Information. Briefly, the as‐prepared amino‐NHC‐coated Au NPs were first centrifuged and then redispersed in a solution containing aniline and sodium dodecyl sulfate (SDS). SDS is known to adsorb onto Au NP surfaces and form a surface‐associated surfactant layer. Aniline can be enriched within this SDS layer due to the strong interactions between aniline and SDS. As a result, the local concentration of aniline near the Au NP interface is expected to be higher than in the bulk solution. Following this step, an ammonium persulfate solution dissolved in an acidic medium was introduced into the mixture, initiating the oxidative polymerization of aniline. This polymerization occurred both on the surface of the Au NPs and within the bulk solution. Due to the differences in density, the unbound PANI could be efficiently removed via centrifugation. Multiple washing steps were performed for thorough purification.

Furthermore, the thickness of the resultant PANI shell could be readily tailored by varying reaction parameters such as aniline concentration, reaction time and polymerization cycles. This approach enabled the preparation of a series of core‐shell nanocomposites with different PANI thicknesses, which are named as Au NP@NHC‐PANI‐1, Au NP@NHC‐PANI‐2, Au NP@NHC‐PANI‐3, Au NP@NHC‐PANI‐4, and Au NP@NHC‐PANI‐5, respectively. For comparative analysis, Au NP@PANI reference nanocomposites (without NHC ligands) were prepared under identical conditions. Such control is crucial for studying the relationship between PANI thickness, colloidal stability, and electrical conductivity, enabling a comprehensive evaluation of how the NHC linker influences the interfacial characteristics and overall performance of the nanocomposite.

Transmission electron microscopy (TEM) was performed to determine PANI thickness, which ranged from 3 ± 1, 6 ± 2, 12 ± 2, 22 ± 3, and 32 ± 3 nm for Au NP@NHC‐PANI‐1, Au NP@NHC‐PANI‐2, Au NP@NHC‐PANI‐3, Au NP@NHC‐PANI‐4, Au NP@NHC‐PANI‐5, respectively. The UV–vis spectra of the Au NP@NHC‐PANIs with varying thicknesses of the PANI shell are presented in Figure 2b. As the thickness of the PANI shell increased, the characteristic absorption bands of PANI at around 420 and 850 nm gradually increased, indicating the progressive formation of the polymer layer. The band centered around 540 nm corresponds to the localized surface plasmon resonance (LSPR) of the Au NPs. With increasing PANI shell thickness, the LSPR peak exhibited a gradual redshift. This shift can be attributed to the increase in the Au NP local dielectric constant, which reduces the restoring force on the collective oscillation of conduction electrons, thereby lowering the resonance energy of the plasmon mode [47].

**FIGURE 3:**
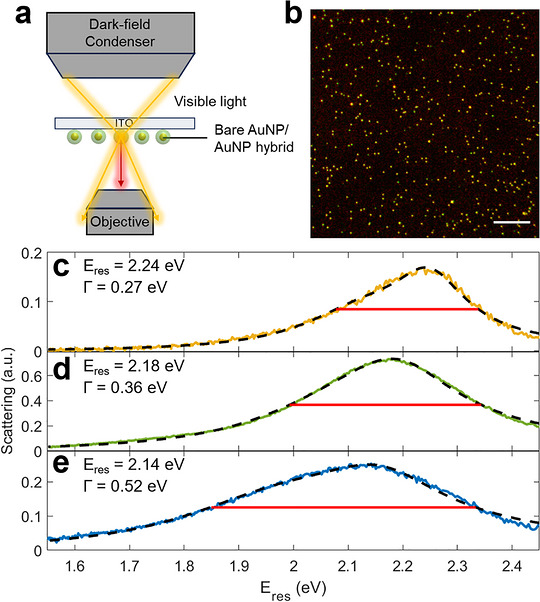
Single‐particle scattering spectroscopy reveals plasmonic properties of nanohybrids at a sub‐population level. (a) Schematic diagram of single‐particle dark‐field microscope used to collect the plasmon scattering of Au NPs and hybrids. (b) Image of the sample field of view. Sample in the image represents uncoated bare Au NPs, scale bar = 10 µm. (c–e) Representative single‐particle scattering spectra of (c) uncoated Au NP; (d) Au NP@PANI‐2; and (e) Au NP@NHC‐PANI‐2. Black‐dashed lines represent Lorentzian fitting for each scattering spectrum. Red solid lines represent the linewidth for each scattering spectrum.

In addition to the shell thickness, electronic interactions between the PANI shell and the Au NP core can also influence the LSPR. Interfacial charge transfer introduces additional non‐radiative decay pathways and modifies the LSPR, providing an effective way to evaluate how NHC linkages influence interfacial charge transport. To gain deeper insight into this effect, single‐particle scattering spectroscopy was employed to directly probe the plasmonic behavior of Au NP@PANI‐2 and Au NP@NHC‐PANI‐2 with both the core diameter and PANI shell thickness kept constant (Figure 3). Compared with the bare Au NPs (Figure 3c, *E*_res_ = 2.24 eV, Γ = 0.27 eV), the Au NP@PANI‐2 (Figure 3d) exhibited a moderate redshift (E_res_ = 2.18 eV) accompanied by a slight linewidth broadening (Γ = 0.36 eV). In contrast, the Au NP@NHC‐PANI‐2 (Figure 3e) displayed a more pronounced red shift (E_res_ = 2.14 eV) together with a substantial increase in linewidth (Γ = 0.52 eV). Linewidth broadening in both cases reflects enhanced non‐radiative damping of the plasmon mode due to charge transfer, suggesting that the NHC linkage promotes stronger electronic coupling across the Au NP@NHC‐PANI interface. The change in medium dielectric constant with both polymer coatings compared to the bare Au NPs is responsible for the moderate plasmon redshift that in turn mainly lowers bulk damping, leading to a slight underestimation in Figure 3 for the linewidth broadening caused by interfacial charge transfer [48, 49, 50].

**FIGURE 4:**
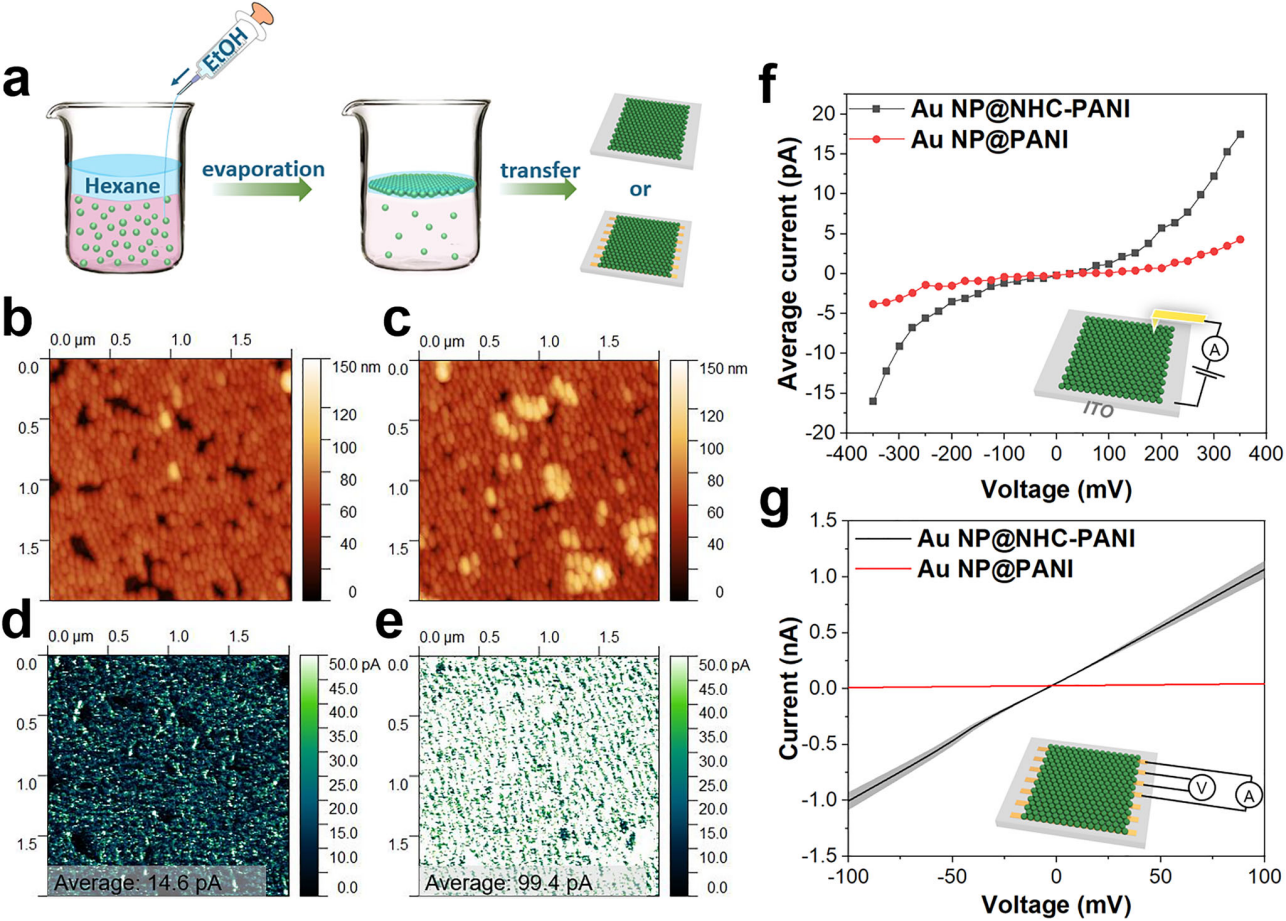
(a) Schematic illustration of the interface‐assisted 2D assembly of Au NPs, enabling transfer onto ITO substrates or pre‐patterned electrodes. (b and c) AFM topography images of Au NP@PANI and Au NP@NHC‐PANI monolayers. (d and e) C‐AFM current maps of Au NP@PANI and Au NP@NHC‐PANI measured under a bias of 300 mV. (f) Current–voltage (*I–V*) curves of Au NP@PANI and Au NP@NHC‐PANI obtained *via* the average current of C‐AFM images at different biases. (g) Lateral conductance of Au NP monolayers on gold electrodes measured using the four‐probe method.

To corroborate the spectroscopic evidence of enhanced interfacial charge transfer, direct electrical measurements were performed to investigate the charge transport properties of Au NP@PANI‐2 and Au NP@NHC‐PANI‐2. To minimize variations due to film thickness or morphology, self‐assembled monolayers (SAMs) were fabricated using an oil–water interfacial self‐assembly method (Figure 4a). Specifically, SAMs of Au NP@PANI‐2 and Au NP@NHC‐PANI‐2 were assembled into a uniform single‐layer film at the water/*n*‐hexane interface by slowly adding ethanol [51, 52]. This technique enabled the formation of large‐area, well‐ordered NP monolayers, as confirmed by atomic force microscopy (AFM) (Figures 4b,c and S17). While minor defects were observed, the overall film quality was sufficient for reliable conductivity analysis.

The resulting monolayer could be easily transferred onto various substrates, for example indium tin oxide (ITO), to enable vertical conductivity characterizations via conductive AFM (C‐AFM). Under a bias voltage of 300 mV, the contact current of the Au NP@PANI‐2 composite was measured to be 14.6 pA (Figure 4d), whereas Au NP@NHC‐PANI‐2 exhibited a significantly higher contact current of 99.4 pA (Figure 4e), demonstrating that the NHC interfacial layer can enhance charge transport between the Au NP and PANI. Furthermore, the average contact currents under various applied voltages were also measured to obtain current–voltage (*I–V*) curves. As shown in Figure 4f, *I*–*V* curves clearly show that incorporating the NHC linker improved the conductance of the SAM film. Moreover, to complement these vertical transport studies, lateral conductance was also measured using the four‐probe method by transferring the SAMs onto pre‐patterned gold electrodes. As shown in Figure 4g, the conductance of Au NP@NHC‐PANI‐2 reached 3.5 nS, more than twenty‐times higher than that of Au NP@PANI‐2 (0.17 nS), further confirming that the NHC linker can efficiently enhance interfacial charge transport.

**FIGURE 5:**
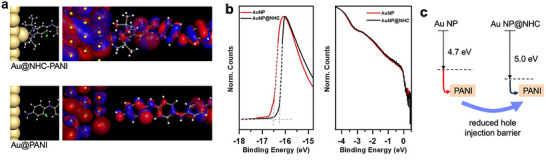
(a) DFT‐optimized structures (left) and corresponding real‐space transmission eigenchannels (right) for Au@NHC‐PANI (top) and Au@PANI (bottom) junctions at low energies. (b) UPS spectra on Au NPs and Au NP@NHC, including the photoemission onset (left) and valence band features (right). (c) energy level diagram illustrating the reduction in the hole injection barrier for Au NP@NHC.

To further demonstrate enhanced interfacial charge transport enabled by the NHC linker, Au@NHC‐PANI and Au@PANI junction models were constructed and their transmission functions were evaluated within a density functional theory (DFT) + non‐equilibrium Green's function (NEGF) framework, allowing us to directly compare electronically coupled, chemisorbed enabling interfaces with physisorbed contacts. The calculated transmission function for the Au@NHC‐PANI junction is much higher than that of the physisorbed Au@PANI junction, indicating stronger electronic coupling across the interface (Figure S19). This behavior is further supported by the real‐space transmission eigenchannels, which show continuous, interface‐spanning conducting pathways for Au@NHC‐PANI, whereas such pathways are absent for Au@PANI contact (Figure 5a). Sulfur‐based contacts (Au@S‐PANI and Au@SH‐PANI) were also modeled. The chemisorbed anchored junction shows higher transmission than the physisorbed contact (Figure S19), consistent with the conclusion that chemisorbed anchoring improves interfacial transport relative to physisorption [53]. A strictly quantitative ranking of different linker chemistries (e.g., NHC vs thiol) in experiment, however, would require additional control of parameters such as surface coverage and protonation state.

To investigate the energy levels of the Au NPs with and without NHC, ultraviolet photoemission spectroscopy (UPS) measurements were conducted. The secondary‐electron cutoff spectra indicate that the work function (WF) increases from 4.70 eV (Au NP) to 5.00 eV (Au NP@NHC) (Figure 5b), while both samples exhibit a clearly metallic nature, evidenced by the pronounced Fermi step in their valence band spectra. The increase in the WF of the Au NP@NHC suggests the formation of a reduced energetic offset for interfacial charge transfer between the Au NP@NHC and PANI (Figure 5c), consistent with the increased currents observed in Figure 4.

Although gold cores are highly conductive, the overall conductivity of the nanocomposite is predominantly determined by the conductivity of the PANI shell. To assess the effect of shell thickness, C‐AFM measurements were performed on samples with increasing PANI thickness. As shown in Figure S17, the contact current of Au NP@NHC‐PANI decreased sharply from 99.4 pA (PANI‐2) to 4.4 pA (PANI‐3) and 0.9 pA (PANI‐4), while Au NP@NHC‐PANI‐1 was excluded from this study due to its aggregation during centrifugation. A comparable trend was observed for Au NP@PANI, where the current decreased from 14.6 pA (PANI‐2) to 2.4 pA (PANI‐3) and was completely unmeasurable (0 pA) for PANI‐4. Besides, electrochemical impedance spectroscopy (EIS) of Au NP@NHC‐PANI‐2 and Au NP@NHC‐PANI‐3 were also carried out (Figure S18). The solution resistances are similar for the two samples (*R*_s_ ≈ 29.8 and 28.7 Ω), whereas the effective polarization resistance increases strongly with shell thickness (*R*_p_ ≈ 227.3 Ω for Au NP@NHC‐PANI‐2 and 3781.1 Ω for Au NP@NHC‐PANI‐3). Moreover, the Bode phase plots show a well‐defined relaxation feature in the intermediate frequency range (10–1000 Hz) with the characteristic peak frequency shifting from 223.9 to 50.1 Hz, together with a change in the low‐frequency phase from approximately −80° (Au NP@NHC‐PANI‐2) to −63° (Au NP@NHC‐PANI‐3). These trends are consistent with an increased transport path length and polarization distribution in the thicker‐shell sample [54]. These findings indicate the importance of rationally designing the PANI thickness to achieve optimal conductivity in hybrid nanocomposites. The electrochemical stability of the Au NP@NHC‐PANI composites was also measured by cyclic voltammetry (CV). As shown in Figures S20 and S21, both Au NP@NHC‐PANI‐2 and Au NP@NHC‐PANI‐3 exhibited stable redox behaviors without obvious decay in peak current after 200 continuous cycles, demonstrating the excellent electrochemical stability of the hybrid nanocomposites.

**FIGURE 6:**
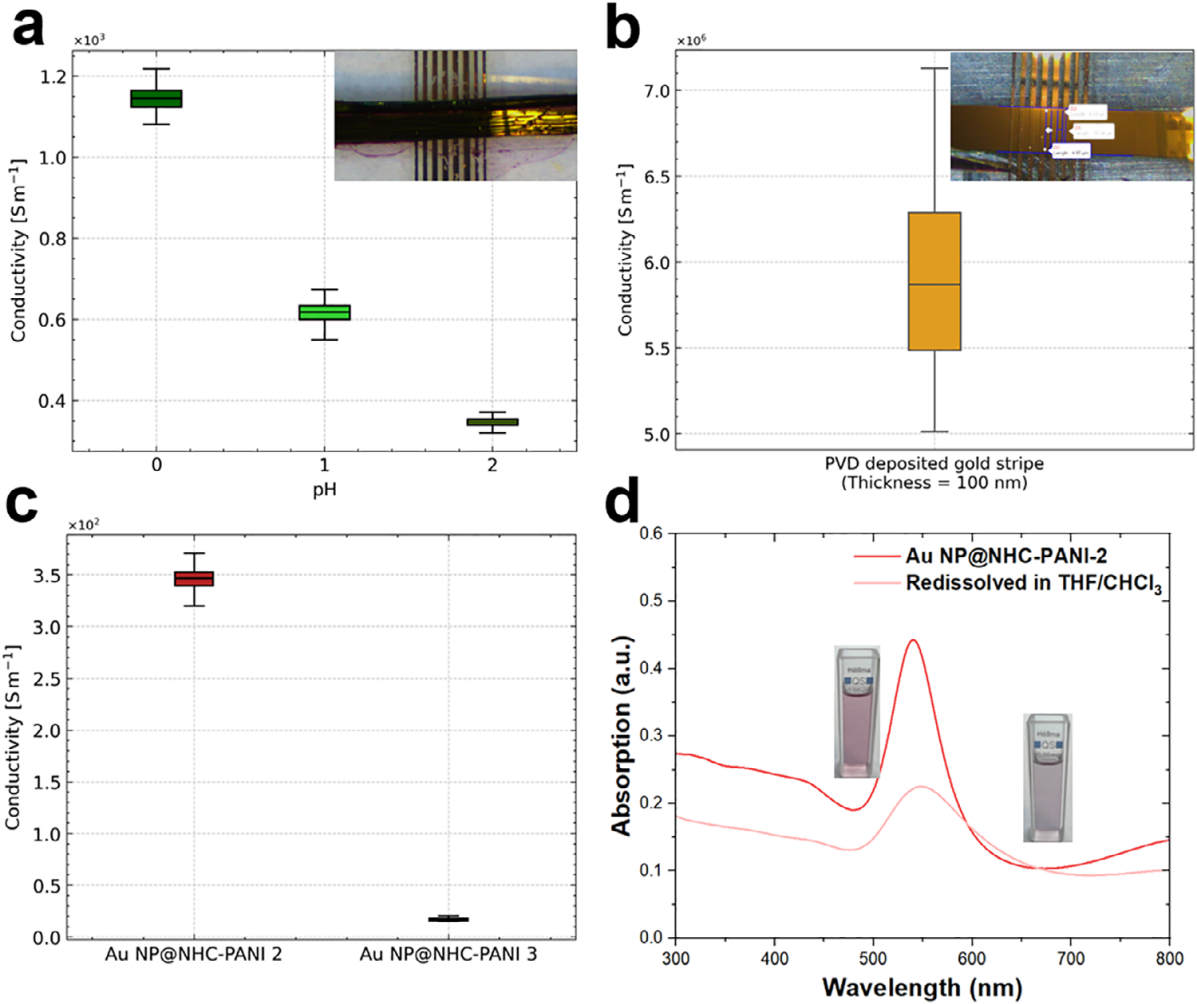
(a) Conductivity of Au NP@NHC‐PANI‐2 at different pH values (0–2). Inset: photograph of Au NP lines coated on pre‐patterned gold electrodes. (b) Conductivity of a thermally deposited gold stripe (thickness 100 nm) by physical vapor deposition (PVD). Inset: photograph of the deposited stripe on pre‐patterned electrodes. (c) Comparison of the conductivity of Au NP@NHC‐PANI‐2 and Au NP@NHC‐PANI‐3 measured at pH 2. (d) UV–vis spectra of Au NP@NHC‐PANI‐2 before and after redissolution in THF/CHCl_3_ (1/3).

To evaluate the application of Au NP@NHC‐PANI for printable electronics, the conductivity of Au NP@NHC‐PANI after acid doping of the PANI shell was investigated. As the electrical properties of PANI are sensitive to doping levels, samples were treated at different pH values, and the conductivity of Au NP@NHC‐PANI‐2 was calculated to be 1.15 × 10^3^ S/m (pH = 0), 6.12 × 10^2^ S/m (pH = 1) and 3.52 × 10^2^ S/m (pH = 2) (Figure 6a). For comparison, a thermally evaporated gold film measured under the same conditions exhibited a conductivity of 5.85 × 10^6^ S/m (Figure 6b), which is approximately three orders of magnitude higher. This reduced conductivity primarily arises from the lower intrinsic conductivity of doped PANI (∼10 S/m) compared to that of bulk gold. Nevertheless, the measured values still represent an improvement over pristine doped PANI. As expected, increasing the thickness of the PANI shell led to a decrease in overall conductivity; for instance, at pH 2, Au NP@NHC‐PANI‐3 exhibited a conductivity of 4.1 S/m, comparable to that of pure PANI (Figure 6c). This result suggests that when the polymer shell becomes sufficiently thick, the contribution of the gold core to charge transport becomes negligible. Although the conductivity of Au NP@NHC‐PANI‐2 is far below that of bulk gold, the hybrid material retains processing advantages. To demonstrate this, printing of Au NP@NHC‐PANI‐2 into the shape of a TUHH lettering pattern was performed as shown in Figure S22. Additionally, an initial ambient‐aging test of the printed electrodes showed a decrease in average conductance from 1.56 to 1.54 S after one week (Figure S23), which can be attributed to slow de‐doping of PANI and/or humidity‐related influence in air. Moreover, owing to the intrinsic solubility of PANI, Au NP@NHC‐PANI‐2 could be redispersed in organic solvents such as THF/CHCl_3_ (Figure 6d), with only a slight red shift observed in the LSPR. This redispersibility enables the preparation of recyclable nanoparticle inks, which hold great promise for sustainable electronic applications. Further optimization of the CP ligands will be necessary to enhance conductivity, colloidal stability and printability, ultimately enabling the development of high‐performance, reusable and room‐temperature‐processable nanoparticle inks.

## 3 Conclusions

In summary, we have demonstrated an in situ polymerization strategy for synthesizing large, NHC‐coupled Au NP‐PANI hybrids that overcome the size, shape, and solvent limitations associated with previous methods. For the first time, we provide direct experimental validation that NHC linkages can effectively enhance interfacial charge transport in Au NP‐CP systems. Single‐particle scattering spectroscopy reveals that NHCs strengthen interfacial electronic coupling, as indicated by pronounced plasmonic linewidth broadening arising from charge‐transfer‐mediated damping. Furthermore, SAMs of Au NP@NHC‐PANI exhibit improved vertical and lateral conductivities compared with their counterparts Au NP@PANI. Together, these results establish that interfacial molecular design using NHCs can bridge the electronic gap between inorganic nanoparticles and organic conductors, opening new opportunities for electronically integrated hybrid nanostructures in nanoelectronic applications.

## Conflicts of Interest

The authors declare no conflicts of interest.

## Supporting information




**Supporting File**: anie72401‐sup‐0001‐SuppMat.docx.

## Data Availability

The data that support the findings of this study are available from the corresponding author upon reasonable request.

## References

[1] V. M. Agranovich , Y. N. Gartstein , and M. Litinskaya , “Hybrid Resonant Organic–Inorganic Nanostructures for Optoelectronic Applications,” Chemical Reviews 111 (2011): 5179–5214. 21780839 10.1021/cr100156x

[2] J. Sun , Y. Choi , Y. J. Choi , et al., “2D–Organic Hybrid Heterostructures for Optoelectronic Applications,” Advanced Materials 31 (2019): 1803831, 10.1002/adma.201803831. 30786064

[3] O. Voznyy , B. R. Sutherland , A. H. Ip , D. Zhitomirsky , and E. H. Sargent , “Engineering Charge Transport by Heterostructuring Solution‐Processed Semiconductors,” Nature Reviews Materials 2 (2017): 17026, 10.1038/natrevmats.2017.26.

[4] X. Ye , C. Zhu , P. Ercius , et al., “Structural Diversity in Binary Superlattices Self‐Assembled from Polymer‐Grafted Nanocrystals,” Nature Communications 6 (2015): 10052, 10.1038/ncomms10052. PMC4686769 26628256

[5] A. M. Steiner , F. Lissel , A. Fery , J. Lauth , and M. Scheele , “Prospects of Coupled Organic–Inorganic Nanostructures for Charge and Energy Transfer Applications,” Angewandte Chemie International Edition 60 (2021): 1152–1175, 10.1002/anie.201916402. 32173981 PMC7821299

[6] R. Ahmad , N. Griffete , A. Lamouri , N. Felidj , M. M. Chehimi , and C. Mangeney , “Nanocomposites of Gold Nanoparticles@Molecularly Imprinted Polymers: Chemistry, Processing, and Applications in Sensors,” Chemistry of Materials Journal 27 (2015): 5464–5478, 10.1021/acs.chemmater.5b00138.

[7] A. Kamyshny and S. Magdassi , “Conductive Nanomaterials for 2D and 3D Printed Flexible Electronics,” Chemical Society Reviews 48 (2019): 1712–1740, 10.1039/C8CS00738A. 30569917

[8] B. Reiser , L. González‐García , I. Kanelidis , J. H. M. Maurer , and T. Kraus , “Gold Nanorods with Conjugated Polymer Ligands: Sintering‐Free Conductive Inks for Printed Electronics,” Chemical Science 7 (2016): 4190–4196, 10.1039/C6SC00142D. 30155064 PMC6014069

[9] A. Escudero , L. González‐García , R. Strahl , D. J. Kang , J. Drzic , and T. Kraus , “Large‐Scale Synthesis of Hybrid Conductive Polymer–Gold Nanoparticles Using ‘Sacrificial’ Weakly Binding Ligands for Printing Electronics,” Inorganic Chemistry 60 (2021): 17103–17113. 34735769 10.1021/acs.inorgchem.1c02350

[10] M. A. H. Klos , L. González‐García , and T. Kraus , “Mechanically Robust, Inkjet‐Printable Polymer Nanocomposites with Hybrid Gold Nanoparticles and Metal‐Like Conductivity,” ACS Applied Material Interfaces 16 (2024): 31576–31585, 10.1021/acsami.4c04692. PMC11195551 38859578

[11] D. J. Kang , Y. Jüttke , L. González‐García , A. Escudero , M. Haft , and T. Kraus , “Reversible Conductive Inkjet Printing of Healable and Recyclable Electrodes on Cardboard and Paper,” Small 16 (2020): 2000928. 10.1002/smll.202000928 32462772

[12] J. Wiklund , A. Karakoç , T. Palko , et al., “A Review on Printed Electronics: Fabrication Methods, Inks, Substrates, Applications and Environmental Impacts,” Journal of Manufacturing and Materials Processing 5 (2021): 89, 10.3390/jmmp5030089.

[13] J. Peng , Q. Lin , T. Földes , et al., “In Situ Spectro‐Electrochemistry of Conductive Polymers Using Plasmonics to Reveal Doping Mechanisms,” ACS Nano 16 (2022): 21120–21128. 36468680 10.1021/acsnano.2c09081 PMC9798863

[14] J. Lawrence , J. T. Pham , D. Y. Lee , Y. Liu , A. J. Crosby , and T. Emrick , “Highly Conductive Ribbons Prepared by Stick–Slip Assembly of Organosoluble Gold Nanoparticles,” ACS Nano 8 (2014): 1173–1179, 10.1021/nn4057726. 24417627

[15] T. Kister , J. H. M. Maurer , L. González‐García , and T. Kraus , “Ligand‐Dependent Nanoparticle Assembly and Its Impact on the Printing of Transparent Electrodes,” ACS Applied Material Interfaces 10 (2018): 6079–6083, 10.1021/acsami.7b18579. 29400942

[16] M. N. Hopkinson , C. Richter , M. Schedler , and F. Glorius , “An Overview of N‐Heterocyclic Carbenes,” Nature 510 (2014): 485–496, 10.1038/nature13384. 24965649

[17] A. V. Zhukhovitskiy , M. J. MacLeod , and J. A. Johnson , “Carbene Ligands in Surface Chemistry: From Stabilization of Discrete Elemental Allotropes to Modification of Nanoscale and Bulk Substrates,” Chemical Reviews 115 (2015): 11503–11532, 10.1021/acs.chemrev.5b00220. 26391930

[18] S. Engel , E.‐C. Fritz , and B. J. Ravoo , “New Trends in the Functionalization of Metallic Gold: from Organosulfur Ligands to N‐Heterocyclic Carbenes,” Chemical Society Reviews 46 (2017): 2057–2075, 10.1039/C7CS00023E. 28272608

[19] C. A. Smith , M. R. Narouz , P. A. Lummis , et al., “N‐Heterocyclic Carbenes in Materials Chemistry,” Chemical Reviews 119 (2019): 4986–5056, 10.1021/acs.chemrev.8b00514. 30938514

[20] Y. Pan , A. Das , F. Glorius , and J. Ren , “Insights into the Surface Chemistry of N‐heterocyclic Carbenes,” Chemical Society Reviews 54 (2025): 4626–4650, 10.1039/D4CS01299B. 40304210

[21] G. Kaur , R. L. Thimes , J. P. Camden , and D. M. Jenkins , “Fundamentals and Applications of N‐heterocyclic Carbene Functionalized Gold Surfaces and Nanoparticles,” Chemical Communications 58 (2022): 13188–13197, 10.1039/D2CC05183D. 36342012

[22] C. M. Crudden , J. H. Horton , I. I. Ebralidze , et al., “Ultra Stable Self‐assembled Monolayers of N‐Heterocyclic Carbenes on Gold,” Nature Chemistry 6 (2014): 409–414, 10.1038/nchem.1891. 24755592

[23] M. J. MacLeod and J. A. Johnson , “PEGylated N‐Heterocyclic Carbene Anchors Designed To Stabilize Gold Nanoparticles in Biologically Relevant Media,” Journal of the American Chemical Society 137 (2015): 7974–7977, 10.1021/jacs.5b02452. 26081724

[24] R. W. Y. Man , C.‐H. Li , M. W. A. MacLean , et al., “Ultrastable Gold Nanoparticles Modified by Bidentate N‐Heterocyclic Carbene Ligands,” Journal of the American Chemical Society 140 (2018): 1576–1579, 10.1021/jacs.7b08516. 29211456

[25] J. F. DeJesus , L. M. Sherman , D. J. Yohannan , et al., “A Benchtop Method for Appending Protic Functional Groups to N‐Heterocyclic Carbene Protected Gold Nanoparticles,” Angewandte Chemie International Edition 132 (2020): 7655–7660, 10.1002/ange.202001440. 32092219

[26] L. Ge , T. K. H. Trinh , C. Li , et al., “Directed Synthesis of Gold Nanoparticle Superstructures Using Self‐Assembling Peptoids Containing Metal‐Bonding N‐Heterocyclic Carbenes,” Nano Letters 25 (2025): 12049–12058, 10.1021/acs.nanolett.5c02998. 40644600 PMC12333405

[27] N. A. Nosratabad , Z. Jin , L. Du , M. Thakur , and H. Mattoussi , “N‐Heterocyclic Carbene‐Stabilized Gold Nanoparticles: Mono‐Versus Multidentate Ligands,” Chemical Materials 33 (2021): 921–933, 10.1021/acs.chemmater.0c03918.

[28] N. Sun , S. Singh , H. Zhang , et al., “Gold Nanoparticles with N‐Heterocyclic Carbene/Triphenylamine Surface Ligands: Stable and Electrochromically Active Hybrid Materials for Optoelectronics,” Advanced Science 11 (2024): 2400752. 38774949 10.1002/advs.202400752 PMC11304275

[29] L. Zhang , Z. Wei , S. Thanneeru , et al., “A Polymer Solution To Prevent Nanoclustering and Improve the Selectivity of Metal Nanoparticles for Electrocatalytic CO_2_ Reduction,” Angewandte Chemie International Edition 131 (2019): 15981–15987, 10.1002/ange.201909069. 31468668

[30] N. Sun , S. Zhang , F. Simon , et al., “Poly(3‐hexylthiophene)s Functionalized with N‐Heterocyclic Carbenes as Robust and Conductive Ligands for the Stabilization of Gold Nanoparticles,” Angewandte Chemie International Edition 60 (2021): 3912–3917, 10.1002/anie.202012216. 33135279 PMC7898828

[31] J. Tao , D. Zheng , Y. Tang , et al., “Polymer Ligands with Multi‐Nitrogen Heterocyclic Carbenes for Enhanced Stability and Reactivity in Nanoparticle Surface Functionalization,” Angewandte Chemie International Edition 137 (2025): e202419640, 10.1002/ange.202419640. 39865453

[32] A. V. Zhukhovitskiy , M. G. Mavros , T. Van Voorhis , and J. A. Johnson , “Addressable Carbene Anchors for Gold Surfaces,” Journal of the American Chemical Society 135 (2013): 7418–7421, 10.1021/ja401965d. 23668242

[33] E. A. Doud , M. S. Inkpen , G. Lovat , et al., “In Situ Formation of N‐Heterocyclic Carbene‐Bound Single‐Molecule Junctions,” 140 (2018): 8944–8949. 10.1021/jacs.8b05184 29969027

[34] D. T. Nguyen , M. Freitag , M. Körsgen , et al., “Versatile Micropatterns of N‐Heterocyclic Carbenes on Gold Surfaces: Increased Thermal and Pattern Stability with Enhanced Conductivity,” Angewandte Chemie International Edition 57 (2018): 11465–11469, 10.1002/anie.201807197. 29952056

[35] M. J. MacLeod , A. J. Goodman , H.‐Z. Ye , H. V.‐T. Nguyen , T. Van Voorhis , and J. A. Johnson , “Robust Gold Nanorods Stabilized by Bidentate N‐Heterocyclic‐Carbene–Thiolate Ligands,” Nature Chemistry 11 (2019): 57–63, 10.1038/s41557-018-0159-8. 30420777

[36] C. Eisen , B. K. Keppler , J. Min Chin , X. Su , and M. R. Reithofer , “Fabrication of Azido‐PEG‐NHC Stabilized Gold Nanoparticles as a Functionalizable Platform,” Chemical Science 15 (2024): 18524–18533, 10.1039/D4SC04112G. 39430936 PMC11487300

[37] A. Inayeh , R. R. K. Groome , I. Singh , et al., “Self‐assembly of N‐Heterocyclic Carbenes on Au(111),” Nature Communications 12 (2021): 4034, 10.1038/s41467-021-23940-0. PMC8241988 34188031

[38] N. L. Dominique , P. Nalaoh , D. M. Jenkins , R. Vaia , K. Park , and J. P. Camden , “One‐Step Functionalization of Gold Nanorods with N‐Heterocyclic Carbene Ligands,” RSC Advances 15 (2025): 5007–5010. 39957825 10.1039/d5ra00754b PMC11826410

[39] Y. Chen and Y. Zhang , “Fluorescent Quantification of Amino Groups on Silica Nanoparticle Surfaces,” Anal Bioanalytical Chemistry 399 (2011): 2503–2509, 10.1007/s00216-010-4622-7. 21243340

[40] S. V. Patwardhan , F. S. Emami , R. J. Berry , et al., “Chemistry of Aqueous Silica Nanoparticle Surfaces and the Mechanism of Selective Peptide Adsorption,” Journal of the American Chemical Society 134 (2012): 6244–6256, 10.1021/ja211307u. 22435500

[41] M. Wróbel , D. M. Cegiełka , A. Asyuda , K. Kozieł , M. Zharnikov , and P. Cyganik , “N‐Heterocyclic Carbenes—The Design Concept for Densely Packed and Thermally Ultra‐stable Aromatic Self‐assembled Monolayers,” Nano Today 53 (2023): 102024.

[42] L. Qi , R. M. Mayall , D. S. Lee , et al., “Energetics and Redox Kinetics of Pure Ferrocene‐Terminated N‐Heterocyclic Carbene Self‐Assembled Monolayers on Gold,” Langmuir 40 (2024): 17367–17377, 10.1021/acs.langmuir.4c01446. 39106183

[43] C. M. Crudden , J. H. Horton , M. R. Narouz , et al., “Simple Direct Formation of Self‐Assembled N‐Heterocyclic Carbene Monolayers on Gold and Their Application in Biosensing,” Nature Communications 7 (2016): 12654, 10.1038/ncomms12654. PMC5025784 27585494

[44] I. Blakey , T. L. Schiller , Z. Merican , and P. M. Fredericks , “Interactions of Phenyldithioesters with Gold Nanoparticles (AuNPs): Implications for AuNP Functionalization and Molecular Barcoding of AuNP Assemblies,” Langmuir 26 (2010): 692–701, 10.1021/la9023162. 19824687

[45] C. Rossner , Q. Tang , O. Glatter , M. Müller , and P. Vana , “Uniform Distance Scaling Behavior of Planet–Satellite Nanostructures Made by Star Polymers,” Langmuir 33 (2017): 2017–2026, 10.1021/acs.langmuir.6b04473. 28170264

[46] S. Chowdhury , G. Hu , I. M. Jensen , et al., “Vibrational Mode Assignment of Diisopropyl Benzimidazolium N‐Heterocyclic Carbenes on Gold,” Journal of Physical Chemistry C 128 (2024): 13550–13557, 10.1021/acs.jpcc.4c02228.

[47] S. Link and M. A. El‐Sayed , “Spectral Properties and Relaxation Dynamics of Surface Plasmon Electronic Oscillations in Gold and Silver Nanodots and Nanorods,” Journal of Physical Chemistry B 103 (1999): 8410–8426, 10.1021/jp9917648.

[48] S. A. Lee and S. Link , “Chemical Interface Damping of Surface Plasmon Resonances,” Accounts of Chemical Research 54 (2021): 1950–1960, 10.1021/acs.accounts.0c00872. 33788547

[49] A. Jones , E. K. Searles , M. Mayer , et al., “Active Control of Energy Transfer in Plasmonic Nanorod–Polyaniline Hybrids,” Physical Chemical Letters 14 (2023): 8235–8243, 10.1021/acs.jpclett.3c01990. 37676024

[50] H. Chen , X. Kou , Z. Yang , W. Ni , and J. Wang , “Shape‐ and Size‐Dependent Refractive Index Sensitivity of Gold Nanoparticles,” Langmuir 24 (2008): 5233–5237, 10.1021/la800305j. 18435552

[51] L. Hu , M. Chen , X. Fang , and L. Wu , “Oil–water Interfacial Self‐assembly: A Novel Strategy for Nanofilm and Nanodevice Fabrication,” Chemical Society Reviews 41 (2012): 1350–1362, 10.1039/C1CS15189D. 22076485

[52] X. Lu , Y. Huang , B. Liu , et al., “Light‐Controlled Shrinkage of Large‐Area Gold Nanoparticle Monolayer Film for Tunable SERS Activity,” Chemical Materials 30 (2018): 1989–1997, 10.1021/acs.chemmater.7b05176.

[53] M. S. Inkpen , Z.‐F. Liu , H. Li , L. M. Campos , J. B. Neaton , and L. Venkataraman , “Non‐Chemisorbed Gold–sulfur Binding Prevails in Self‐assembled Monolayers,” Nature Chemistry 11 (2019): 351–358. 10.1038/s41557-019-0216-y 30833721

[54] J. Bisquert , “Influence of the Boundaries in the Impedance of Porous Film Electrodes,” Physical Chemistry Chemical Physics 2 (2000): 4185–4192, 10.1039/b001708f.

