# Supplementary material for: Enhancing Interfacial Charge Transport in Gold Nanoparticle@Polyaniline Hybrids via N‐Heterocyclic Carbene Linkers
Source: Angew Chem Int Ed Engl. 2026 May 11;65(26):e26136. doi: 10.1002/anie.202526136 (PMC13285468; doi:10.1002/anie.202526136)
Supplement: Supplementary file 1 — Supporting File: anie72401‐sup‐0001‐SuppMat.docx. [file ANIE-65-e26136-s001.docx]

Supporting information for

**Enhancing Interfacial Charge Transport in Gold Nanoparticle@Polyaniline Hybrids *via* N-Heterocyclic Carbene Linkers**

*Ningwei Sun^1*^, Haoran Zhang^1,2^, Ziwei Zhou^1^, Po Yuen Ho^1^, Ilka Hermes^1^, Yanfei Gao^1,2^, Shivam Singh^3,4^, Dmitry A. Ryndyk^1,5^, Olga Guskova^1^, Zhenyang Jia^6^, Tathagata Chatterjee^7^, Antoine E. Jimenez^8^, Kaline Pagnan Furlan^8^, Marina Sebastian^1^, Christian Rossner^1,9^, Stephan Link^7,10,11^, Christy F. Landes^6,7,10^, Yana Vaynzof^3,4^, Andreas Fery^1,12^ and Franziska Lissel^1,2*^*

^1^Leibniz Institute of Polymer Research Dresden, Hohe Straße 6, Dresden, 01069, Germany

^2^Hamburg University of Technology, Kasernenstraße 12, Hamburg, 21073, Germany

^3^Chair for Emerging Electronic Technologies, TUD Dresden University of Technology, Dresden, 01062, Germany

^4^Leibniz Institute for Solid State and Materials Research Dresden, Dresden, 01069, Germany

^5^Chair for Theoretical Chemistry, TUD Dresden University of Technology, Dresden, 01062, Germany

^6^Department of Chemical and Biomolecular Engineering, University of Illinois Urbana-Champaign, Urbana, IL 61801, USA.

^7^Department of Chemistry, University of Illinois Urbana-Champaign, Urbana, IL 61801, USA.

^8^Karlsruhe Institute of Technology (KIT), Institute for Applied Materials (IAM), Ceramic Materials and Technologies, Karlsruhe, 76131, Germany

^9^University of Chemistry and Technology Prague, Technická 5, Prague 6 166 28, Czech Republic.

^10^Department of Electrical and Computer Engineering, University of Illinois Urbana-Champaign, Urbana, IL 61801, USA.

^11^Materials Research Laboratory, University of Illinois Urbana-Champaign, Urbana, IL 61801, USA.

^12^Chair for Physical Chemistry of Polymeric Materials, TUD Dresden University of Technology, Dresden, 01062, Germany

N. Sun and H. Zhang contributed equally to this work

E-mail: [sun@ipfdd.de; franziska.lissel@tuhh.de](mailto:sun@ipfdd.de;%20franziska.lissel@tuhh.de)

**Methods and Materials**

## Nuclear Magnetic Res**o**nance (NMR) Spectroscopy

A Bruker Avance III 500 spectrometer was used to record ^1^H NMR spectra at 500 MHz and ^13^C NMR spectra at 126 MHz. The spectra were referenced to the residual solvent signals (CDCl_3_: δ(^1^H) = 7.26 ppm, DMSO-*d_6_*: δ(^1^H) = 2.50 ppm). The following abbreviations were used for ^1^H NMR spectra data as listed: s - singlet, d - doublet, dd - doublet of doublet, t – triplet and m -multiplet.

## Ultraviolet-visible (UV/Vis) Spectroscopy

UV-vis spectra were measured on UV/Vis/NIR spectrophotometer Cary 5000 (Agilent Technologies Deutschland GmbH).

**Raman Spectroscopy**

Spectra were obtained using a Renishaw inVia reflex equipped with a stigmatic single-pass spectrometer, gratings of 1200 or 1800 grooves mm^-1^, a Peltier-cooled CCD detector, a HeNe laser (633 nm, 6 mW) as excitation lines by using either a 5x (Zeiss N PLAN, numerical aperture (NA) 0.12, working distance (WD) 14 mm) or a 10x objective lens (Zeiss, NA 0.2).

## Cyclic voltammetry (CV)

Electrochemical analyses were carried out on Autolab PGSTAT302N workstation. Cyclic voltammetry (CV) measurements were carried out in a 3-electrode setup with platinum counter electrode and Ag/AgCl reference electrode. 0.1 M NaCl/HCl was used as the electrolyte. Au NP@PANI coated on ITO substrates were used as working electrodes.

## Transmission Electron Microscopy (TEM)

TEM images were obtained using a Zeiss Libra 120 with an accelerating voltage of 120 kV. Samples were prepared by placing a 2 µL droplet of the diluted NP dispersions or the assembly film on TEM grids (Cu, 200 Mesh, coated with carbon film; Science Services GmbH).

**Single-particle dark-field spectroscopy**

Single-particle dark-field spectroscopy was performed on a custom-built optical setup consisting of an inverted microscope (Zeiss Axio Observer D1m). Illumination was provided by a halogen lamp (Zeiss HAL 100), and the excitation light was directed onto the sample plane through a dark-field condenser (NA 0.7 to 1.4). The scattered light from individual particles was collected in a transmitted geometry using an air objective (Zeiss EC Epiplan-Neofluar 50×, NA = 0.8) and then spatially filtered by a 20 μm mechanical slit before entering a spectrometer (Princeton Instruments Acton SP2150i) equipped with a diffraction grating (500 nm blaze, 300 lines/mm). The dispersed spectra were detected by a CCD camera (Princeton Instruments PIXIS 400). The spectrograph–camera assembly was mounted on a motorized translation stage, allowing sequential acquisition across the field of view and construction of a hyperspectral data cube.

##

## Conductive Atomic Force Microscopy (C-AFM)

AFM measurements were performed in air on a Bruker ICON (Santa Barbara, CA, USA) using a Silicon cantilever with a conductive Platinum Iridium coating (Model: ElectriMulti75-G (BudgetSensors, nominal spring constant: 3 N/m, nominal tip radius: 25 nm) conductive PtIr) in PeakForce Tapping mode at different tip sample voltages. To compensate for the instrument specific potential offset, an additional +7.5 mV was applied to each of the scans. The ITO substrates were electrically connected to the bias voltage using a double-sided copper adhesive. For the current voltage ramps, we performed 29 consecutive full image scans on each of the samples, while varying the voltage between –350 and + 350 mV in 25 mV steps and plotted the average current extracted in the data analysis software Gwyddion.

## Fluorescence spectroscopy

Fluorescence measurements were performed using an Agilent Cary Eclipse spectrofluorometer. Samples were excited at 380 nm, and emission spectra were recorded over the range of 450–600 nm using a slit width of 5 nm.

**Electrochemical impedance spectroscopy (EIS)**

EIS measurements were performed in a three-electrode configuration using an Autolab potentiostat (FRA32 module). The working electrode (WE) was indium tin oxide (ITO) coated with the Au NP@NHC-PANI films. An Ag/AgCl reference electrode and a Pt counter electrode were employed. The electrolyte consisted of 0.5 M NaCl and 0.01 M HCl. Before impedance measurements, the samples were immersed in the electrolyte for 2 hours to ensure full wetting of the NHC-PANI shell. Additionally, the open-circuit potential (OCP) of each sample was monitored until the potential drift was below 3 mV in 15 min, indicating quasi-steady-state conditions. EIS spectra were recorded at the OCP with an AC perturbation amplitude of 5 mV over a frequency range of 10^6^ Hz to 0.1 Hz. The obtained spectra were validated using Kramers-Kronig (KK) consistency tests, confirming the reliability and linearity of the measurements.

For the electrochemical circle fit, the fitting window was objectively defined from the characteristic RC region identified in the Bode phase plots, centered around the phase extremum corresponding to the dominant relaxation process. The fitting was performed using the NOVA software package (Metrohm Autolab). Because the full spectra contain additional contributions beyond the main semicircle, global fitting with a single Randles circuit would be non-unique; therefore, only the dominant RC regime was fitted to extract a comparable effective interfacial polarization resistance.

**X-ray and ultraviolet photoemission spectroscopy (XPS/UPS)**

The samples were transferred to an ultrahigh vacuum chamber (ESCALAB 250Xi by Thermo Scientiﬁc, base pressure: 1 × 10^−10^ mbar) for XPS and UPS characterization. XPS measurements were carried out using an XR6 monochromated Al k𝛼 source (h𝜐 = 1486.6 eV) using a spot size of 650 μm. UPS measurements were carried out using a double-diﬀerentially pumped He discharge lamp (h𝜐 = 21.22 eV) with a pass energy of 2 eV and a bias at −5 V.

## Printing of Au NP@NHC-PANI-2

Additive Manufacturing combined with Colloidal Assembly (AMCA) was used to fabricate a lettering pattern (TUHH) by using a custom-built direct writing machine, where an Au NP@NHC-PANI-2 ink was printed onto quartz substrates (Crystec GmbH). This process is based on meniscus-guided self-assembly at the printing tip, comprised by a 26s-gauge straight needle directly connected to the ink reservoir, a 25 µL-high precision glass-tight syringe (Hamilton Company, 1702 RN). Prior to printing, the ink was centrifuged to remove the surfactant (SDS), to enable proper meniscus formation between needle tip and substrate. Ultrasonic sonication was used to further homogenize the ink for 15 min, prior to loading it into the syringe. High-precision linear stages (Physics Instruments, M-126.2S1) controlled the substrate motion, setting the writing velocity (V_w_) to 15 µm s^-1^, while the syringe plunger extruded the ink at a constant dispense velocity (V_d_) of 20 µm s^-1^. A camera (Edmund Optics, EO-10012C ½’’ CMOS) monitored the printing in situ while the process was managed via macros executed with GCS command (Physics Instruments, GCS Commands).

**Synthesis protocols of the NHC-NH_2_ anchor**

**Synthesis of 5-Boc-amino-benzoimidazole 1 (Boc-Protection):**

In a 100 mL two-neck round-bottom flask, 5-aminobenzimidazole (3.99 g, 30 mmol, 1.5 equiv.) and potassium carbonate (2.78 g, 20 mmol, 1.0 equiv.) were dissolved in 12 mL of DMF and cooled to 0 °C. After stirring for 15 min to ensure complete dissolution, a solution of di-tert-butyl dicarbonate (4.36 g, 20 mmol, 1.0 equiv.) in 6 mL of DMF was added dropwise over 30 min. The ice bath was then removed, and the mixture was stirred overnight at room temperature. Upon completion, the reaction was quenched with water and concentrated under reduced pressure to remove DMF. The crude product was extracted with chloroform (washed three times with water), dried over anhydrous MgSO₄, and filtered. After solvent removal, the residue was purified by column chromatography (DCM/MeOH, gradient from 40:1 to 15:1), affording the Boc-protected product (**1**) as a white powder (0.98 g, 21%). The relatively low yield is mainly attributed to the formation of side products arising from competing Boc protection at different nitrogen sites: Boc substitution at the benzimidazole NH, or simultaneous protection at both the benzimidazole NH and the exocyclic amino group (see dashed arrows in Figure S5). ^1^H NMR (500 MHz, DMSO-*d_6_*, δ): 12.40 – 12.04 (s, 1H), 9.34 – 9.07 (s, 1H), 8.14 – 8.04 (s, 1H), 7.83 – 7.73 (s, 1H), 7.52 – 7.38 (d, 1H), 7.27 – 7.09 (dd, 1H), 1.53 – 1.46 (s, 9H).

**Synthesis of 5/6-Boc-amino-1-isopropyl-benzimidazole 2**

5-Boc-amino-benzoimidazole (0.42 g, 1.80 mmol, 1 eq.) and cesium carbonate (0.93 g, 2.86 mmol, 1.6 eq.) were added to a 100 mL two-neck round bottom flask. Acetonitrile (18 mL) was then added to the flask, and the mixture was stirred for 15 minutes to ensure complete dissolution of the reagents. Subsequently, 2-bromopropane (0.66 g, 5.41 mmol, 3 eq.) was added dropwise to the reaction mixture. The reaction was then heated to reflux at 85 °C and stirred overnight under a nitrogen atmosphere. After cooling down to room temperature, the solvent was removed using a rotary evaporator, leaving behind a residual solid. The solid product was dissolved in dichloromethane and filtered over Celite to remove any remaining impurities. The solvent was then evaporated, yielding a grey solid. NMR spectroscopic analysis revealed that the product consisted of two isomers, with a ratio of 46:54 based on integration values. (0.46 g, yield: 93%). ^1^H NMR (500 MHz, DMSO-*d_6_*, δ): 9.38 – 9.14 (d, 1H), 8.24 – 8.16 (d, 1H), 7.90 – 7.73 (d, 1H), 7.52 – 7.46 (dd, 1H), 7.33 – 7.28 (dd, 0.5H), 7.16 – 7.11 (dd, 0.5H), 4.71 – 4.57 (m, 1H), 1.53 – 1.50 (dd, 6H), 1.50 – 1.47 (s, 9H).

**Synthesis of 5-Boc-amino-1,3-diisopropyl-benzimidazolium bromide 3**

5/6-Boc-amino-1-isopropyl-benzimidazole (0.2105 g, 0.765 mmol, 1 eq.), 2-bromopropane (0.56 g, 4.59 mmol, 6 eq.), and 2 mL of tetrahydrofuran (THF) were mixed in a pressure vessel. The reaction mixture was heated at 85 °C and allowed to proceed for 24 hours. After completion of the reaction, the mixture was carefully transferred to a 100 mL round bottom flask and the solvent, along with any unreacted 2-bromopropane, was removed by evaporation. The crude product was subjected to purification by column chromatography using a dichloromethane (DCM) and methanol (MeOH) mixture in a ratio of 20:1 as the eluent. The resulting brown crystals were collected with a yield of 26% (0.09 g). The low isolated yield is mainly attributed to side reactions, since 2-bromopropane not only alkylates the N of benzimidazole ring as desired, but can also attack the amide-type N of the Boc-protected group, leading to undesired by-products that complicate purification (see dashed arrows in Figure S7). ^1^H NMR (500 MHz, DMSO-*d_6_*, δ): 9.90 – 9.77 (s, 1H), 9.67 – 9.61 (s, 1H), 8.27 – 8.15 (s, 1H), 8.05 – 7.99 (d, 1H), 7.60 – 7.53 (dd, 1H), 5.03 – 4.86 (m, 2H), 1.68 – 1.56 (dd, 12H), 1.55 – 1.46 (s, 9H)

**Synthesis of (5-Boc-amino-1,3-diisopropyl-benzimidazolium)gold bromide 4**

5-Boc-amino-1,3-diisopropyl-benzimidazolium bromide (0.10 g, 0.26 mmol, 1 eq.), Au(SMe_2_)Cl (0.08 g, 0.28 mmol, 1.1 eq.), K_2_CO_3_ (0.18 g, 1.28 mmol, 5 eq.), and 5 mL of anhydrous acetone were added to a two-neck round flask. The mixture was then heated to 65 °C and stirred overnight to promote the formation of the desired product. After cooling to room temperature, the solvent was evaporated, and the residual solid was dissolved in chloroform and filtered over Celite. The solvent was removed under vacuum and the crude product was purified by column chromatography using a gradient eluent (from pure DCM to DCM: MeOH= 40:1) to yield a white solid. (0.09 g, yield: 42%) ^1^H NMR (500 MHz, Chloroform-*d*, δ): 8.10 – 8.03 (s, 1H), 7.52 – 7.47 (d, 1H), 7.08 – 7.03 (dd, 1H), 6.67 – 6.59 (s, 1H), 5.52 – 5.38 (m, 2H), 1.80 – 1.67 (dd, 12H), 1.58 -1.52 (s, 9H).

**Synthesis of** **(5-amino-1,3-diisopropyl-benzimidazolium) gold bromide 5 (Boc-Deprotection)**

To a 10 mL round bottom flask, 2 mL of a 1.25 M hydrogen chloride – methanol solution and (5-Boc-amino-1,3-diisopropyl-benzimidazolium)gold bromide (0.09 g, 0.15 mmol) were added. The flask was then subjected to reflux at 65 °C for 4 hours. The solvent was subsequently removed using a rotary evaporator, and a sodium bicarbonate water solution was added to neutralize the generated hydrochloric acid. After evaporating the water in vacuo, 20 mL of chloroform was added to dissolve the product. The solution was then filtered over a sand core funnel, and the funnel was flushed with chloroform (3 x 10 mL). The solvent was evaporated to obtain a pale-yellow solid product (0.07 g) with a yield of 87%. ^1^H NMR (500 MHz, Chloroform-*d*, δ): 7.40 – 7.34 (d, 1H), 6.86 – 6.82 (d, 1H), 6.73 – 6.67 (dd, 1H), 5.46 – 5.32 (m, 2H), 3.97 – 3.75 (s, 2H), 1.73 – 1.63 (dd, 12H). ^13^C NMR (125 MHz, Chloroform-*d*, δ): 177.86, 143.64, 143.60, 133.68, 125.56, 113.67, 113.05, 97.68, 54.24, 54.07, 53.83, 53.66, 29.69, 21.76, 21.72, 21.53, 21.48.

**Synthesis of Au NP@NHC-PANI**

**Synthesis of Au NPs**

Au NPs with different sizes and ligands were synthesized according to the reported methods.^[S1,S2]^

For the synthesis of citrate-stabilized Au NPs, a seeded growth method by using sodium citrate as the reductant was adopted. Briefly, a solution of 2.2 mM sodium citrate in Milli-Q water (150 mL) was heated for 15 min at 100 °C under vigorous stirring. Then, fresh 1 mL of HAuCl_4_ (25 mM) was injected. After 10 min, the color of the solution changed from yellow to soft pink, and the temperature was reduced to 95 °C. Then, HAuCl_4_ (1 mL, 25 mM) was injected twice into the solution at an interval of 30 min till the size of the nanoparticles reached ~50 nm.

For the synthesis of hexadecyltrimethylammonium chloride (CtaC)-stabilized Au NPs, a seed-mediated growth method was used. First, to synthesize a 2 nm seed, aqueous solutions of hexadecyltrimethylammonium bromide (CtaB, 100 mM, 9.4 mL) and HAuCl_4_ (2.5 mM, 18.4 μL) were mixed and stored at 35 °C for 10 min. Then, 600 μL of a freshly prepared NaBH_4_ (10 mM) solution was added quickly under vigorous stirring. Stirring was stopped after 30 s, and the seeds were then stored for 25 min at 40 °C to completely remove excess NaBH_4_. Second, to synthesize an 8 nm seed, aqueous solutions of CtaC (200 mM, 40 mL), ascorbic acid (AA) (1 M, 6 mL), and the initial CtaB-capped Au clusters (5 mL) were mixed in a 100 mL beaker. An aqueous HAuCl_4_ solution (1.0 mM, 40 mL) was then quickly injected under stirring. The reaction was allowed to continue at room temperature for 15 min. Third, aqueous solutions of CtaC (50 mM, 200 mL), AA (1 M, 780 μL), and 8 nm CtaC-capped seeds (500 μL) were mixed. In a separate vessel, 400 mL of a growth solution including HAuCl_4_ (1.5 mM) and CtaC (55 mM) was prepared and equilibrated at 45 °C. This growth solution was added dropwise using a syringe pump system at an injection rate of 0.5 mL/min, and the reaction was allowed to continue for 12 h at room temperature after the injection was finished. The final product was collected by centrifugation at 400 rcf for 30 min and washed twice using a 2 mM CtaC solution. The surface ligands (CtaC) could subsequently be exchanged with citrate through a sequential ligand exchange process using SDS as an intermediate.

**Ligand exchange of Au NPs by NHC-Au complex**

According to the reported method,^[S3,S4]^ the ligand exchange process to obtain NHC-functionalized Au NPs was conducted by introducing an NHC solution to displace the original ligands on the Au NPs. Briefly, a 10 mL aqueous solution of citrate-stabilized Au NPs was placed in a glass vial, and an aliquot of the NHC-Au complex stock solution (4 μL, 1 mM in acetonitrile) was added under ambient conditions. The mixture was thoroughly mixed for 10 minutes under ambient conditions, allowing the NHC ligands to replace the citrate ligands on the surface of the Au NPs, leading to the formation of NHC-functionalized aqueous Au NPs. During this process, careful control over the amount of the added NHC ligand was essential to ensure that the Au NPs form a stable colloidal solution. To further purify and isolate the NHC-Au NPs, the resulting nanoparticle solution underwent centrifugation for 30 minutes. After the centrifugation, the aqueous supernatant was carefully removed, leaving behind the purified and isolated NHC-Au NPs, which were characterized and used for PANI coating.

**Calculation of NHC ligand on one Au NP**

The number of NHC ligands per Au NP was estimated based on the experimental conditions:

Total gold content in Au NP solution: A 25 mM HAuCl₄ solution (31.22 mL) corresponds to 0.1537 g of Au:

$$0.025Mol/L \times0.03122L \times196.97g/Mol = 0.1537g$$

The final Au NP dispersion volume was 957.53 mL, giving an Au concentration:

$$0.1537 g \div0.95753 L=0.16052 g/L$$

5 mL Au NP includes Au:

$$0.16052 g/L\times0.05L=8.026\times{10}^{-4}g$$

50 nm Au NP volume:

$$V=\frac{4\pi r^{3}}{3}=6.5450\times{10}^{4} nm^{3}=6.5450\times{10}^{-17} cm^{3}$$

50 nm Au NP mass:

$$6.5450\times{10}^{-17}cm^{3}\times19.3 g/cm^{3}= 1.2632\times{10}^{-15}g$$

Number of Au NPs in 5 mL solution:

$$\frac{8.026\times{10}^{-4}g}{1.2632\times{10}^{-15}g}=6.3537\times{10}^{11}$$

Number of NHC ligands added:

For 5 mL Au NPs, 2 μL of 1 mM NHC-Au complex in acetonitrile corresponds to:

$$2\times{10}^{-6} L\times1\times{10}^{-3} mol/L=2 \times{10}^{-9} mol$$

This equals NHC molecule number:

$$2\times{10}^{-9} mol\times6.02\times{10}^{23} mol^{-1}=1.204\times{10}^{15}$$

Therefore, NHC per Au NP:

$$1.204\times{10}^{15}\div6.3537\times{10}^{11}=1.895\times{10}^{3}$$

**Surface coverage determination by fluorescence spectroscopy**

A dispersion of Au NPs of size 50 nm (1 mL, Au concentration = 0.3 mM) was centrifuged at 14000 x g for 45 minutes. The concentrated Au NP dispersion was then added to 1 μL of 2 mM NHC ligand solution in acetonitrile, diluted in 1mL of water. After ligand grafting, the Au@NHC were isolated by centrifugation (14000 x g, 2 h).

The unbound NHC ligands obtained after a single step of centrifugation (2 hours, 14000 x g) were reacted with 10 mM fluorescamine dye diluted in acetone (15x in excess) ^[S5]^ and subsequently analyzed using fluorescence spectroscopy. The fluorescence signal was integrated over the wavelength range of 470 – 600 nm to determine the concentration of unbound NHC ligands using a calibration curve generated from known NHC concentrations reacted with fluorescamine.

**Figure S1:** Fluorescence emission spectra of fluorescamine-reacted NHC molecules used for quantitative analysis of unbound NHC concentration.

**Constructing the Langmuir adsorption isotherm**

Initially, a calibration curve of fluorescence intensity vs. NHC concentration was obtained (Figure S2).

**Figure S2:** Fluorescence calibration curve of fluorescamine-reacted NHC ligands at different concentrations, used for quantitative determination of unbound NHC. The fluorescence signal was integrated over a wavelength range of 470 nm to 600 nm.

Next, NHC molecules were grafted to Au NPs at varying feed concentrations. After surface grafting and removal of nanoparticles by centrifugation, the concentration of NHC molecules in the supernatant was determined, and from this (knowing the feed concentration) the concentration of NHC molecules at the Au NPs surface (which can be represented in units of molecules per nm^2^). The obtained data were fit to a Langmuir isotherm of adsorption:

$\frac{c}{Ƭ}=\frac{c}{Ƭ_{\max}}+\frac{1}{K\cdotƬ_{\max}}$ (eq. 1)

In this equation, $Ƭ$ is the surface grafting density, $Ƭ_{\max}$ is the surface grafting density at saturation, and *K* is the adsorption equilibrium constant.

From a fit of the experimental data to eq. 1 (Figure S3), the grafting density at saturation was determined to be approx. 4.0 ligands per nm^2^ and the adsorption equilibrium constant was estimated as 8.6 nM^-1^, corresponding to a Gibbs free energy of adsorption of -56 kJmol^−1^ at 21°C, which indicates a strong driving force for ligand exchange of initial citrate against NHC molecules.

**Figure S3:** Linearized Langmuir isotherm (c/ Ƭ vs. c) for NHC adsorption on Au nanoparticles.

**PANI polymerized on NHC stabilized Au NPs**

The PANI was polymerized over Au NPs through a chemical oxidative polymerization method. Initially, a concentrated solution of NHC stabilized Au NPs (corresponding to 5 mL of the original dispersion) was mixed with aniline (2.5 mL, 2 mM) and SDS (0.5 mL, 40 mM). This mixture was vigorously shaken for 30 seconds, and subsequently, ammonium persulfate (APS, 2.5 mL, 2 mM in 10 mM HCl) was added to initiate the oxidative polymerization of aniline. Once the polymerization was complete, the resulting Au NP@NHC-PANI composites were purified by repeated centrifugation and redispersion in 4 mM SDS to remove free PANI not bound to the nanoparticles. By adjusting polymerization parameters (e.g., reaction time, monomer ratio and multiple cycles), it is possible to obtain various Au NP@NHC-PANI structures with different PANI thicknesses. Control samples of Au NP@PANI were synthesized following the same protocol.

**Assembly of** **Au NP@NHC-PANI**

The Au NP@NHC-PANI monolayer film was carefully prepared using a surface-assisted assembly method. Briefly, the Au NP@NHC-PANI solution (2.5 mL) was combined with deionized water (2.5 mL) in a glass culture dish (6 cm in diameter). Then, 4 mL of hexane was slowly introduced to the aqueous solution, forming an oil-water interface. Subsequently, ethanol (5 mL) was gently injected into the aqueous solution at a rate of ~0.1 mL/min. Following the injection, the culture dish was covered with a glass slide, carefully controlling the slow evaporation of hexane. The resulting superlattice monolayer film was then delicately deposited onto transparent ITO or pre-patterned gold substrates. After the deposition, the film was allowed to dry, and then the film was subjected to multiple thorough rinses with water, resulting in the clean Au NP@NHC-PANI monolayers on different substrates. The assembly of Au NP@PANI was carried out in the same condition.

**Experimental Data**

Nuclear Magnetic Resonance (NMR) Spectroscopy


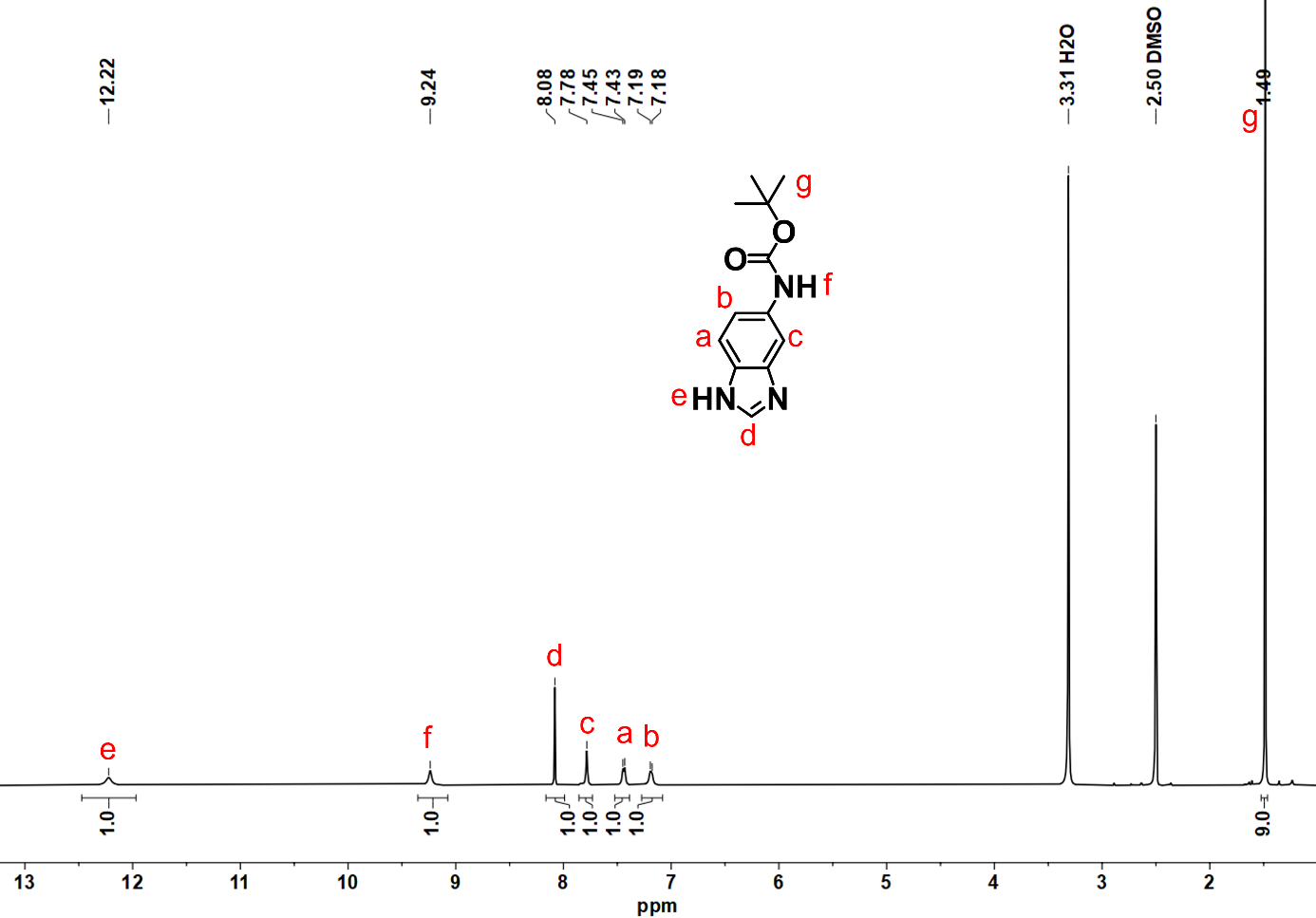


**Figure S4**: ^1^H-NMR spectrum of 5-Boc-amino-benzoimidazole (**1**).


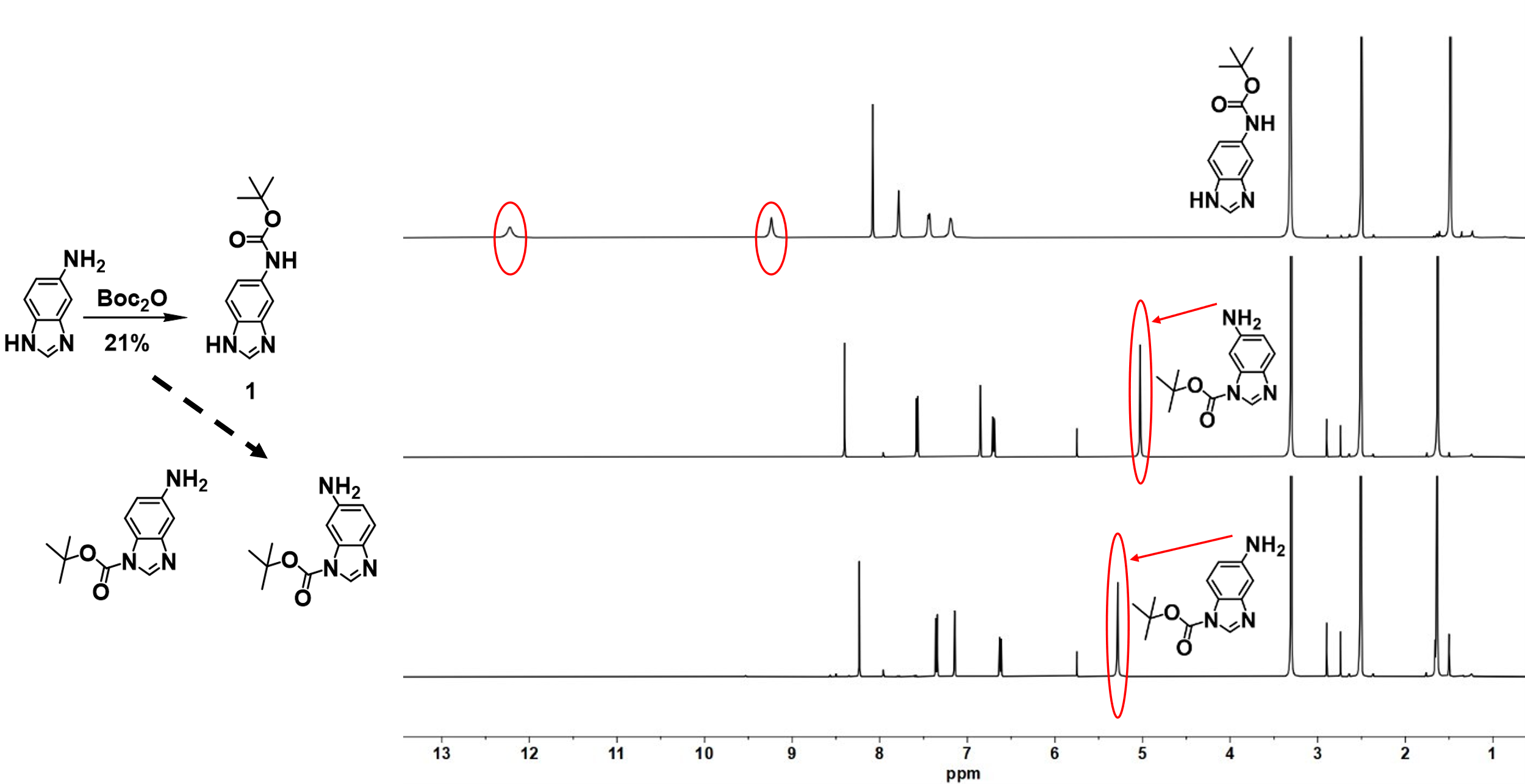


**Figure S5**: Side products in the synthesis of **1** and the corresponding NMR spectra.


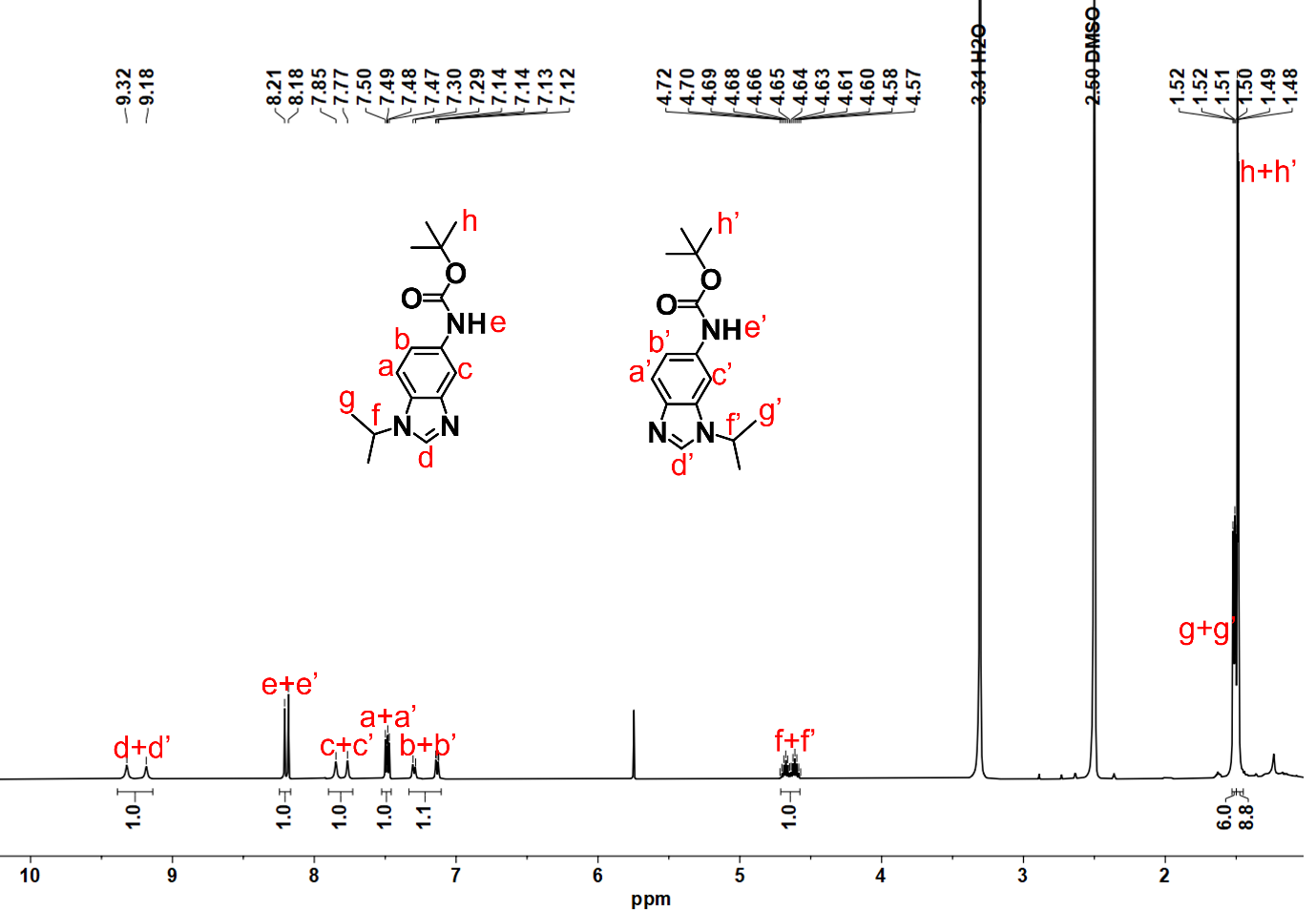


**Figure S6:** ^1^H-NMR spectrum of 5/6-Boc-amino-1-isopropyl-benzimidazole (**2**).


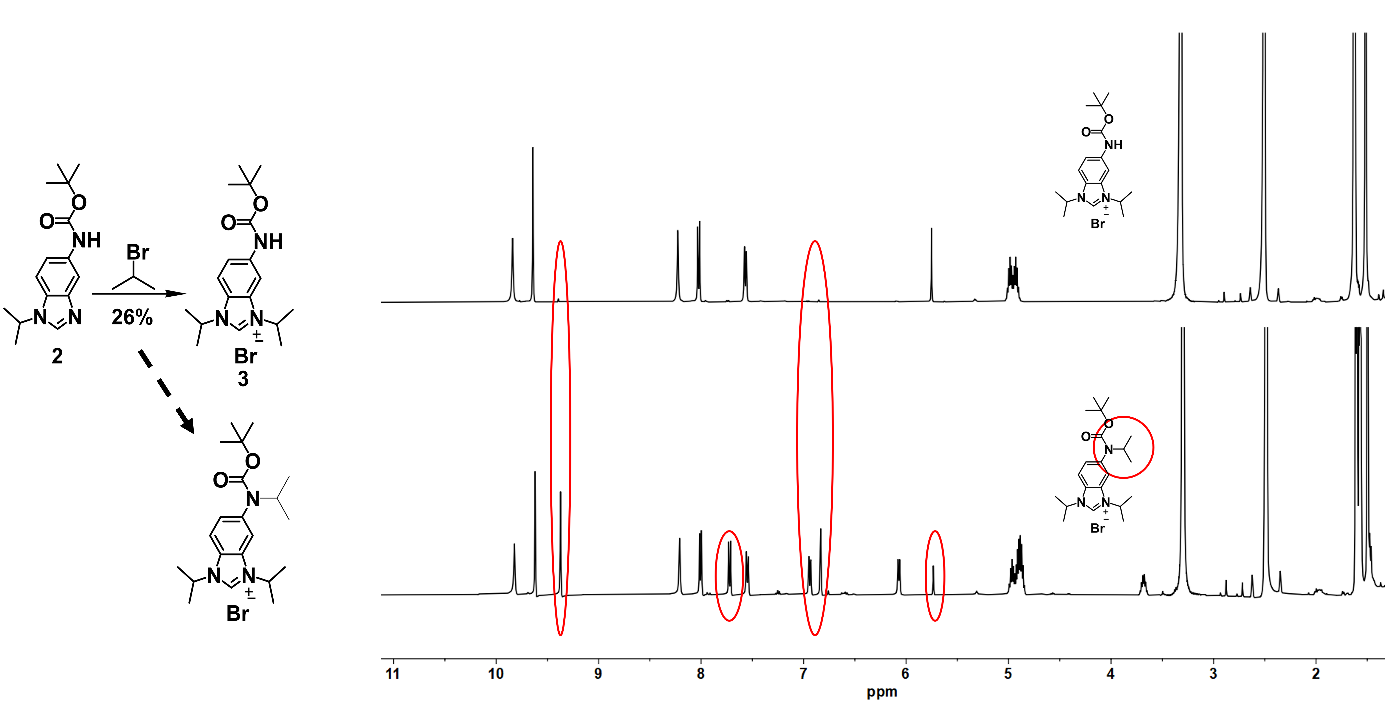


**Figure S7**: Side products in the synthesis of **2** and the corresponding NMR spectra.


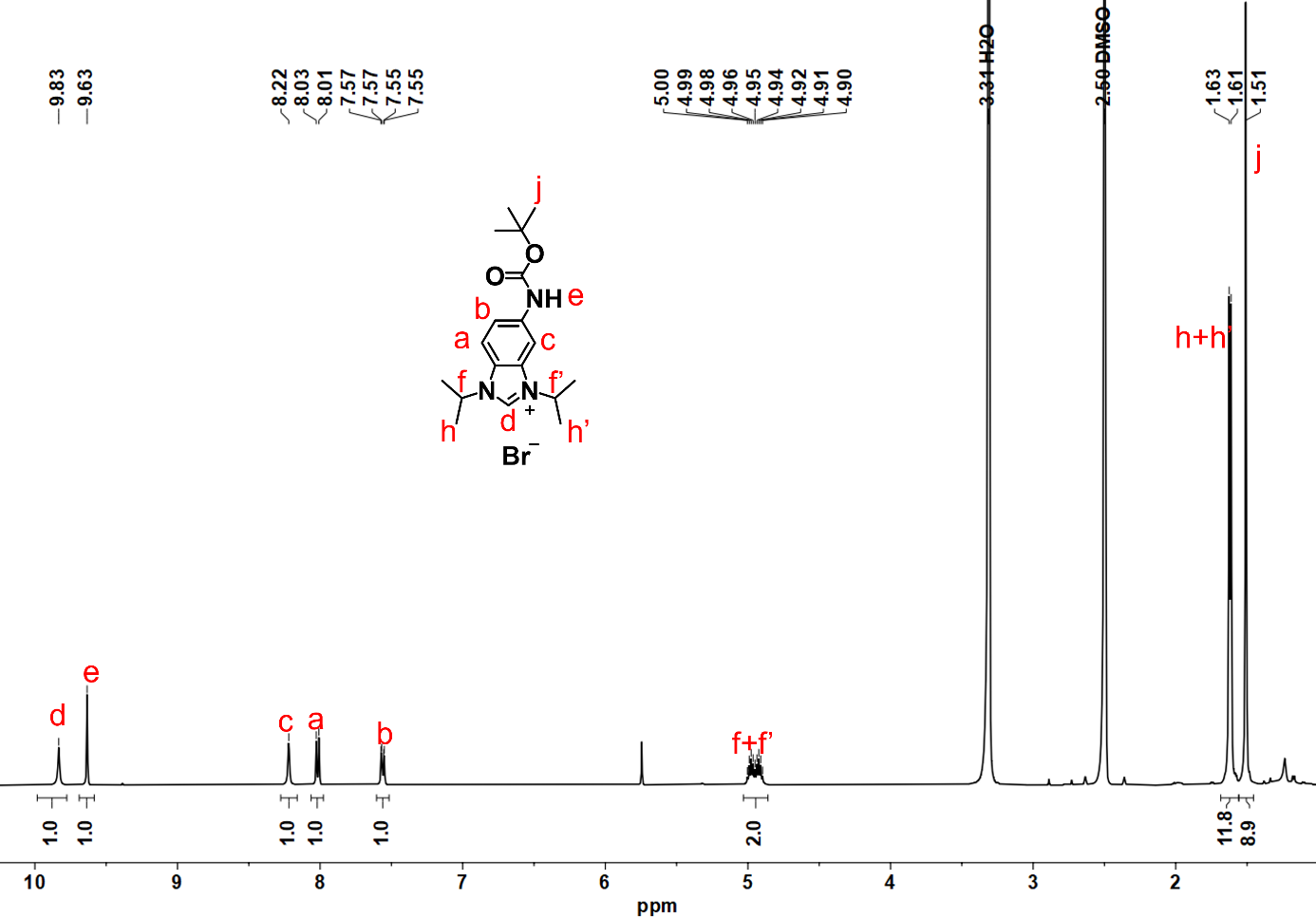


**Figure S8**: ^1^H-NMR spectrum of 5-Boc-amino-1,3-diisopropyl-benzimidazolium bromide (**3**).


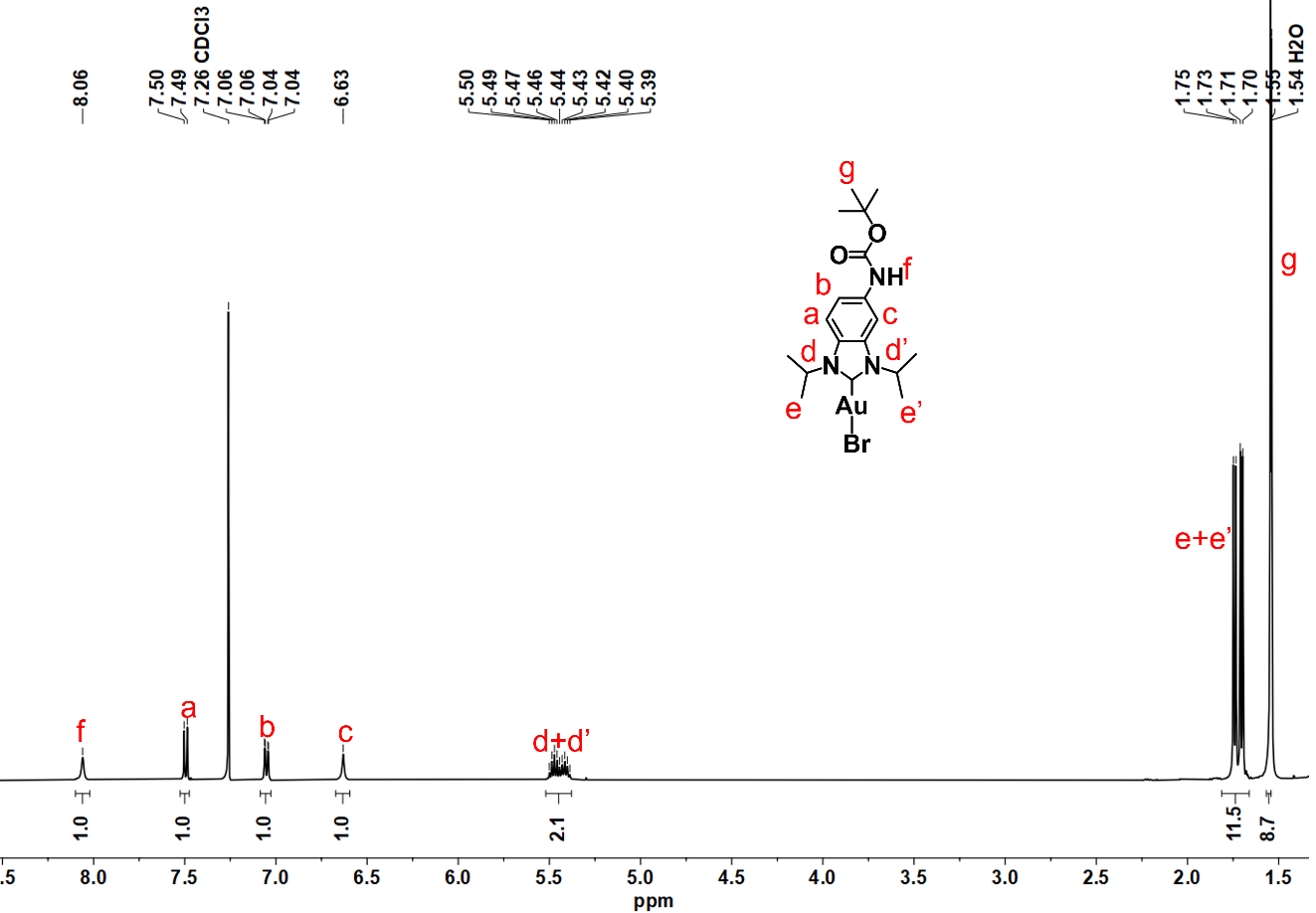


**Figure S9**: ^1^H-NMR spectrum of (5-Boc-amino-1,3-diisopropyl-benzimidazolium)gold bromide (**4**).


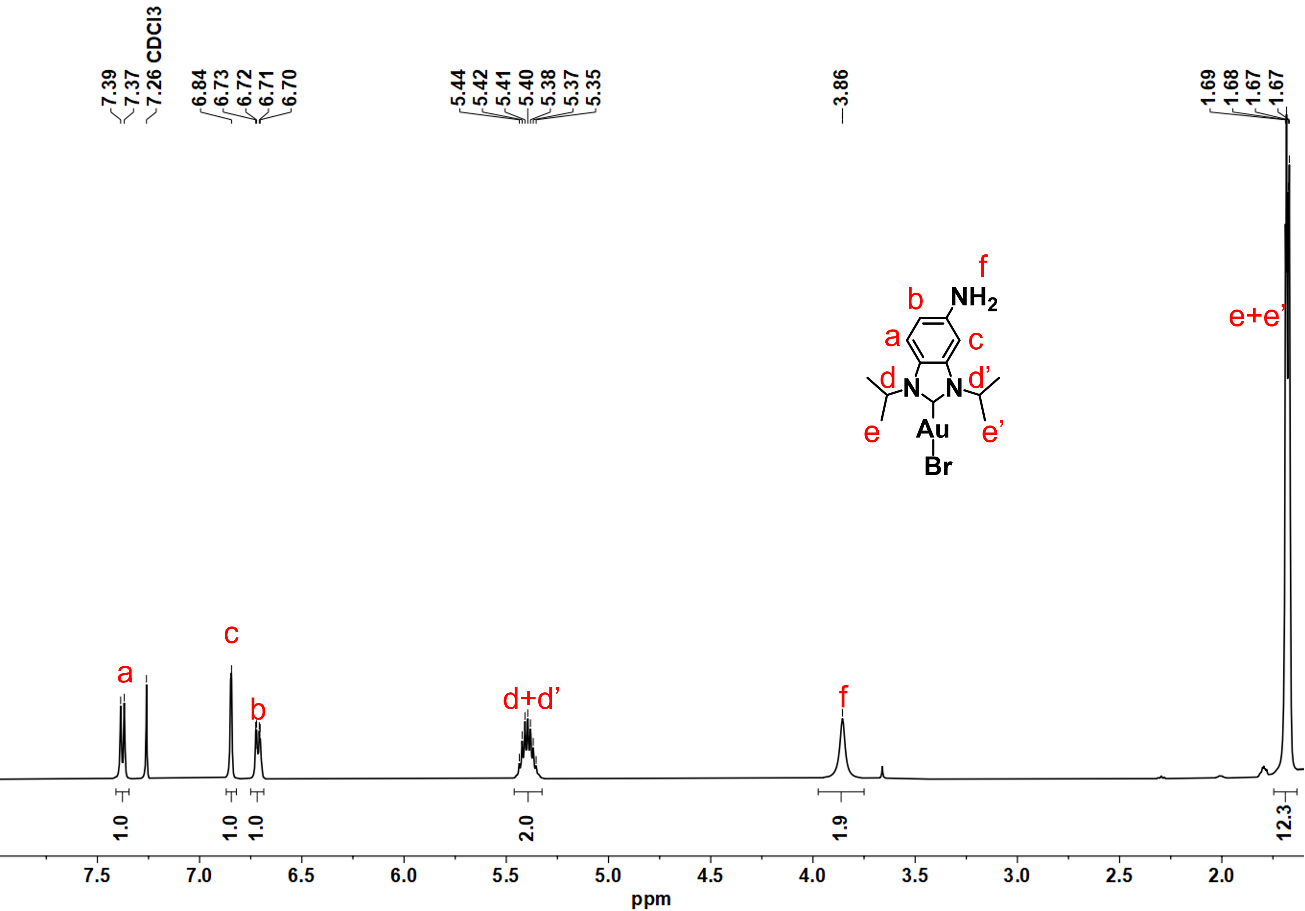


**Figure S10**: ^1^H-NMR spectrum of (5-amino-1,3-diisopropyl-benzimidazolium)gold bromide (**5**).


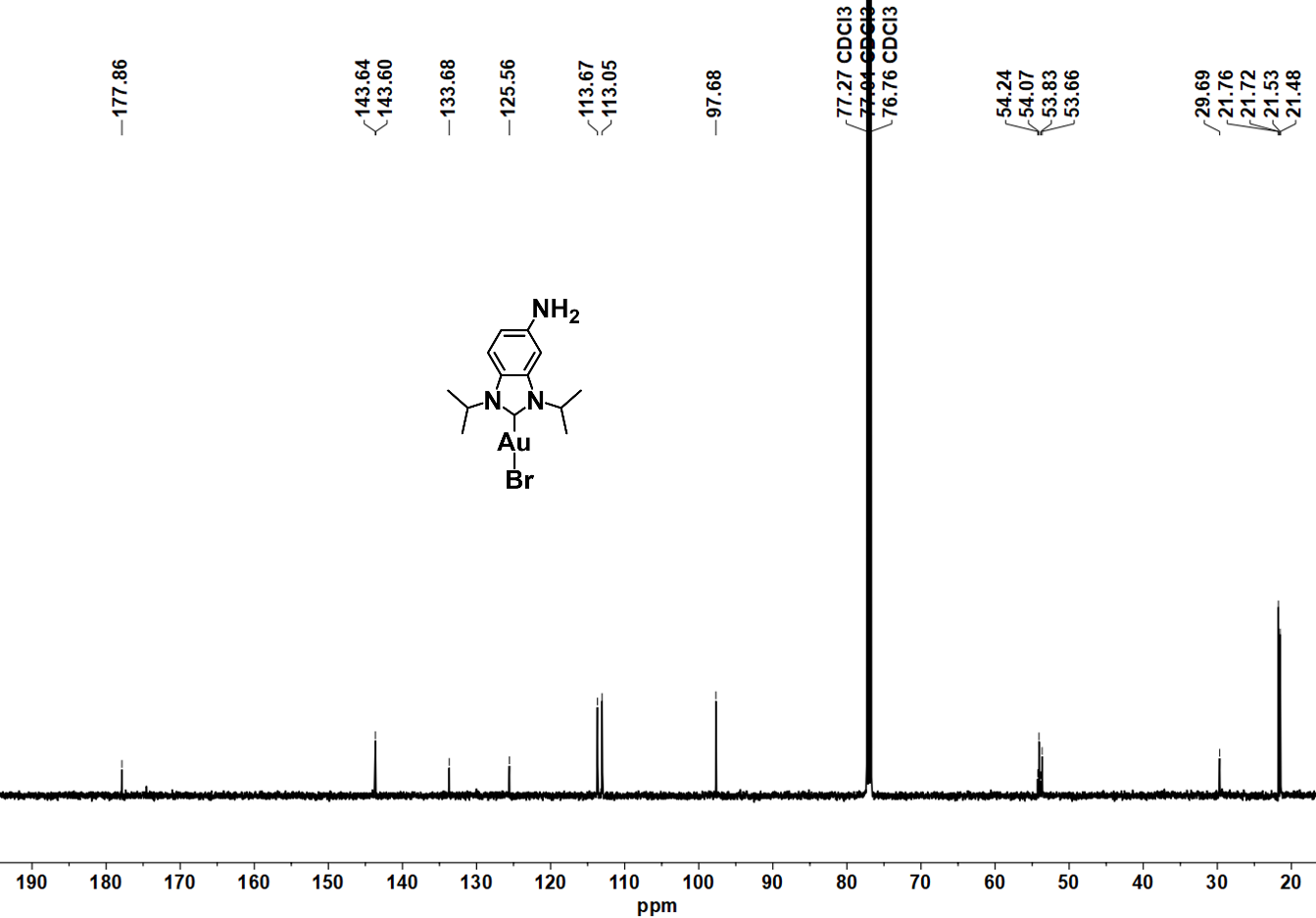


**Figure S11**: ^13^C-NMR spectrum of (5-amino-1,3-diisopropyl-benzimidazolium)gold bromide (**5**).


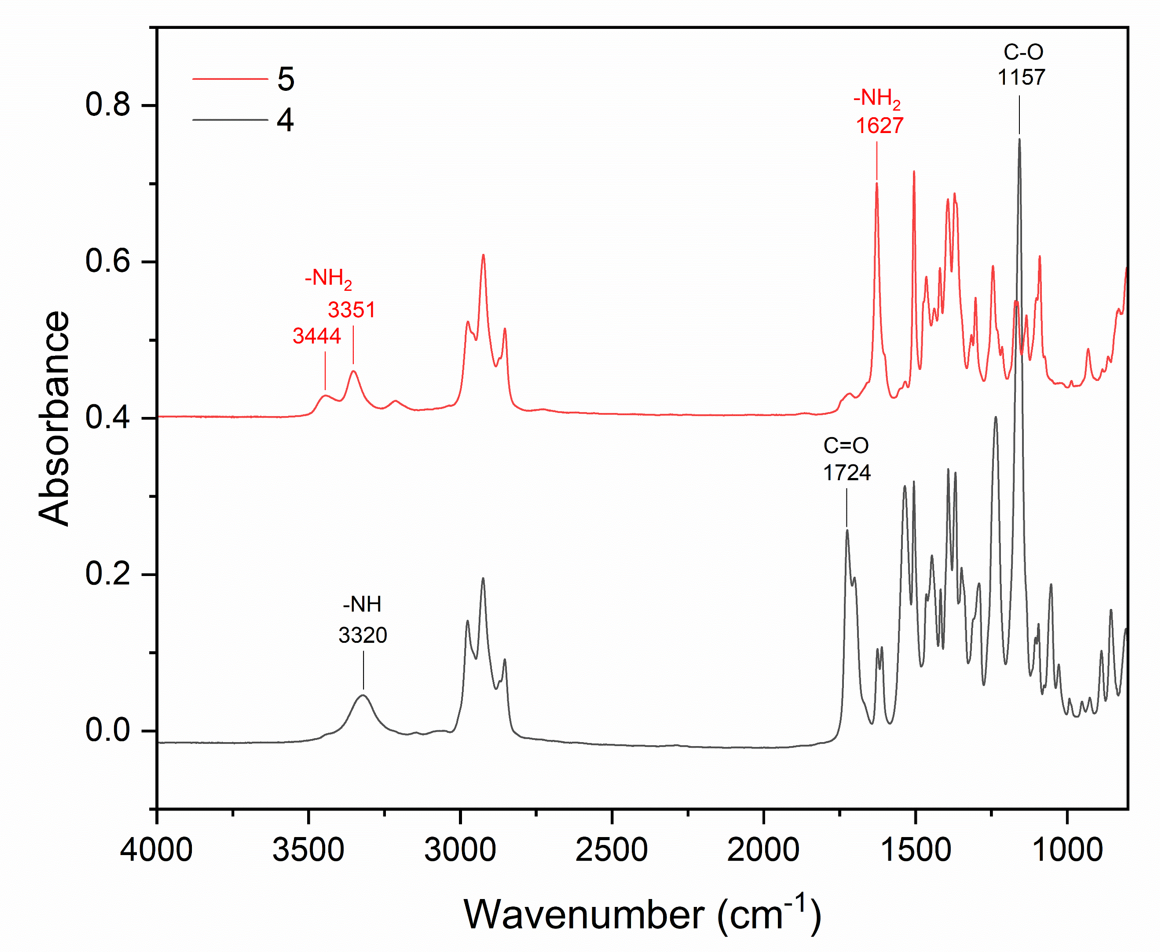


**Figure S12**. FTIR spectra of **4** and **5**.


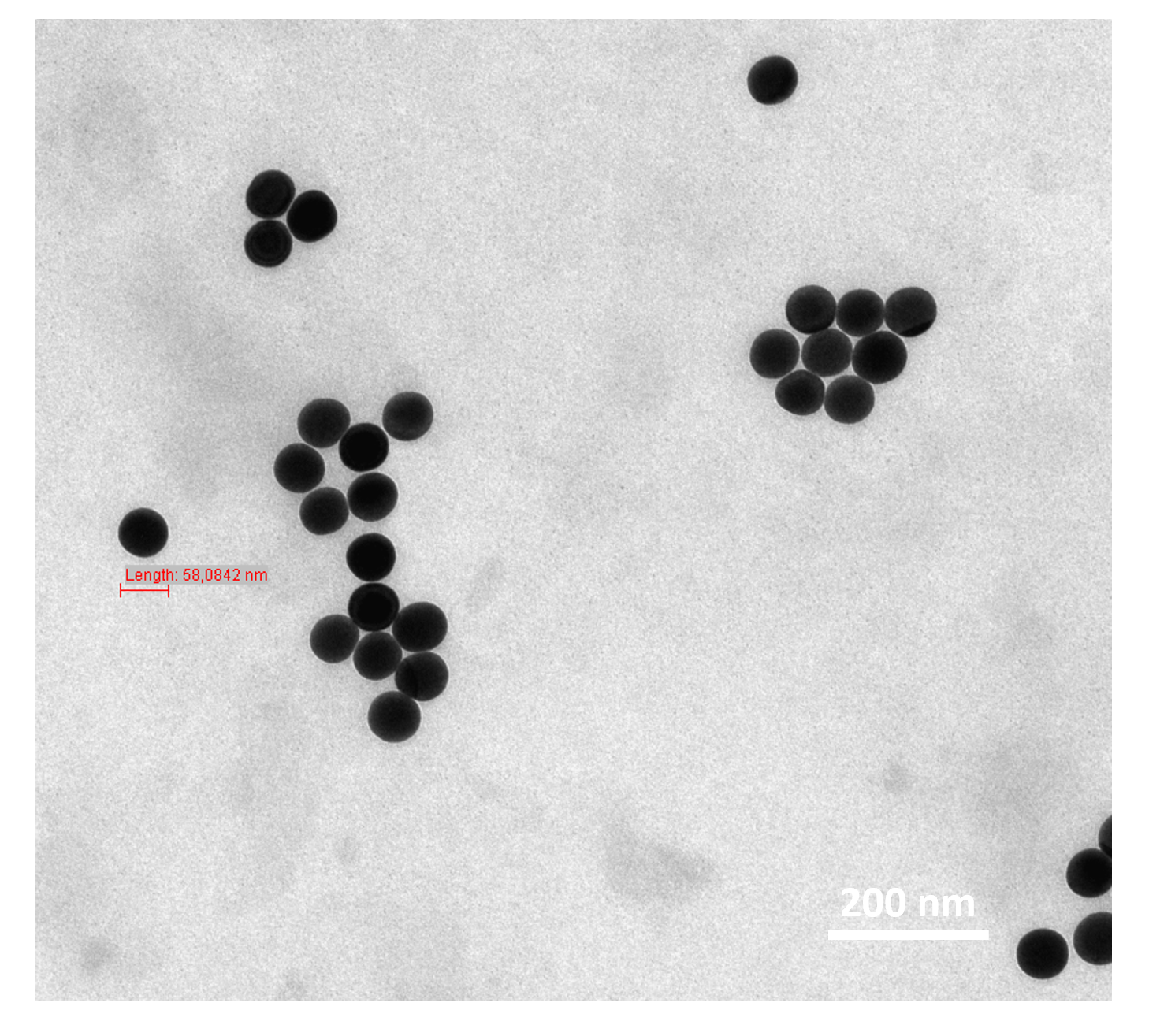


**Figure S13**. TEM image of the prepared CtaC stabilized Au NPs.


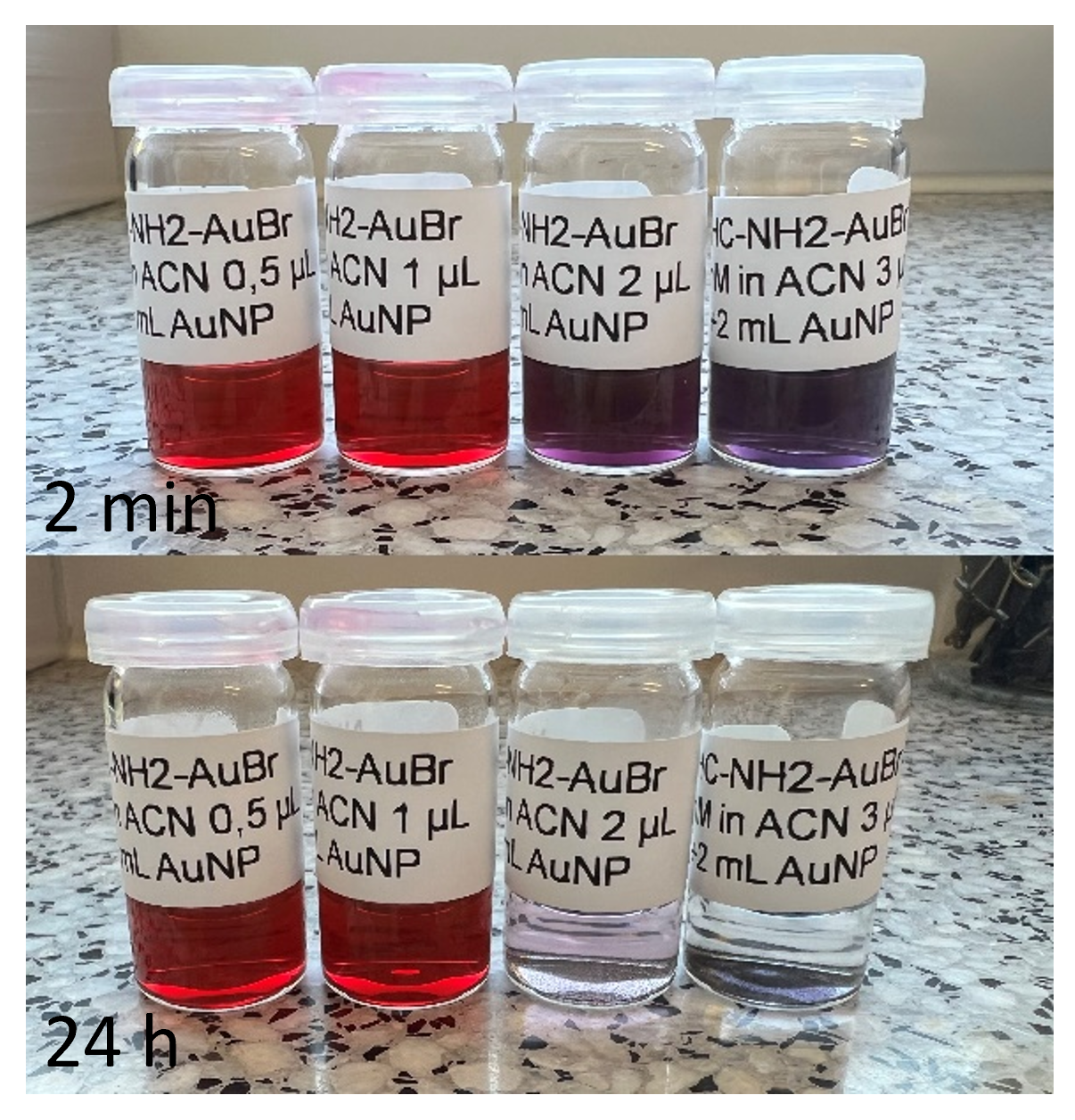


**Figure S14**. Photographs of Au NP dispersions after addition of different amounts of **5** (0.5, 1, 2, and 3 μL; 1 mM in acetonitrile) to 2 mL of Au NP solution, recorded after 2 min (top) and 24 h (bottom).


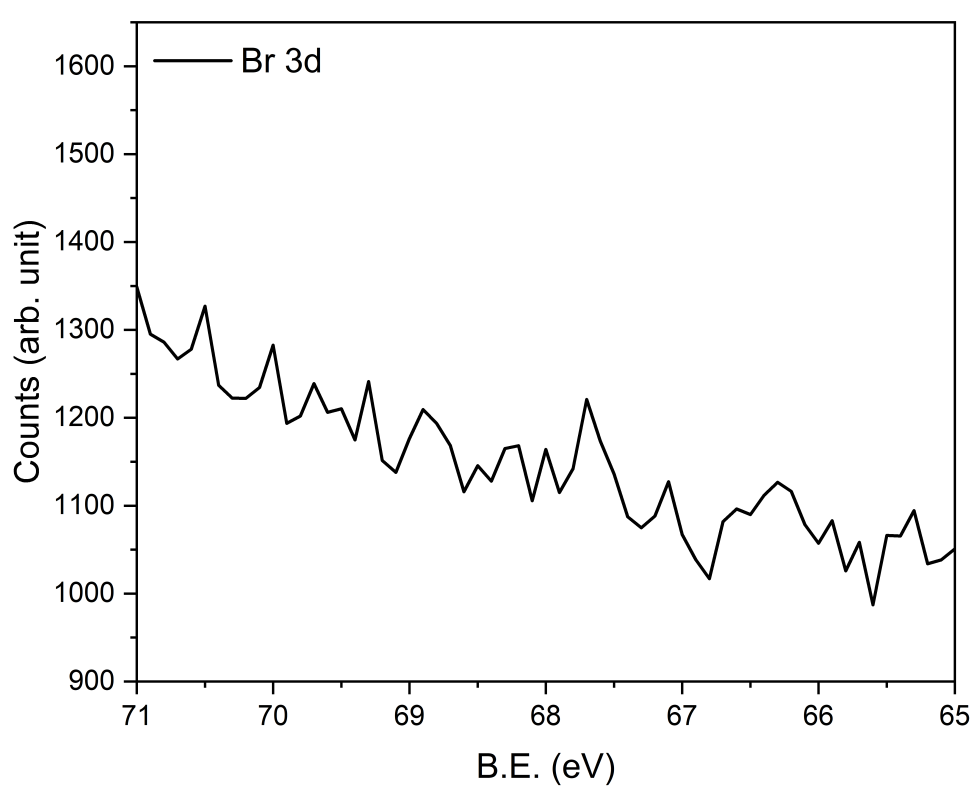


**Figure S15.** X-ray photoelectron spectroscopy (XPS) Br 3d region of Au NP@NHC after ligand exchange and washing/purification. No distinct Br 3d signal is observed within the detection limit, indicating negligible Br residue on the Au NP surface.


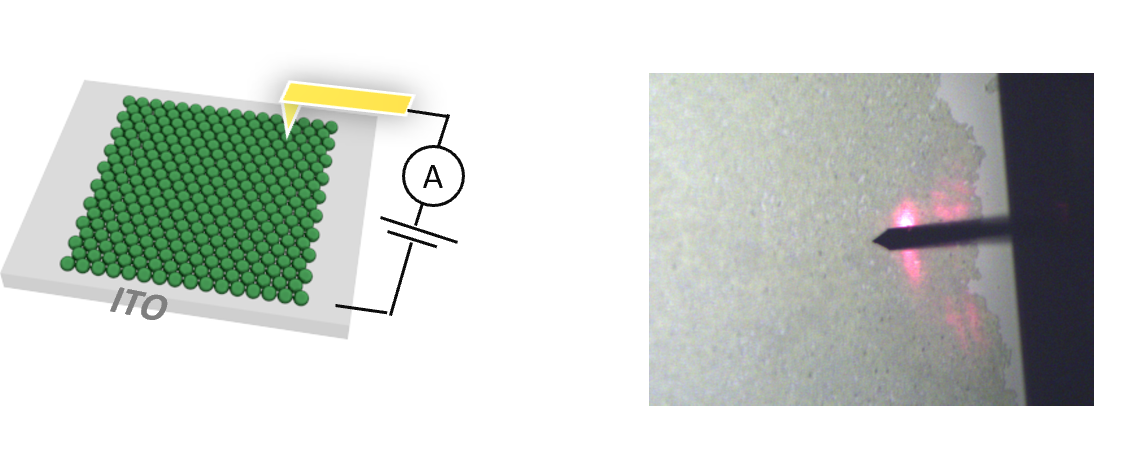


**Figure S16.** Schematic illustration (left) and optical image (right) of the C-AFM measurement setup used for characterizing SAMs assembled on ITO substrates.


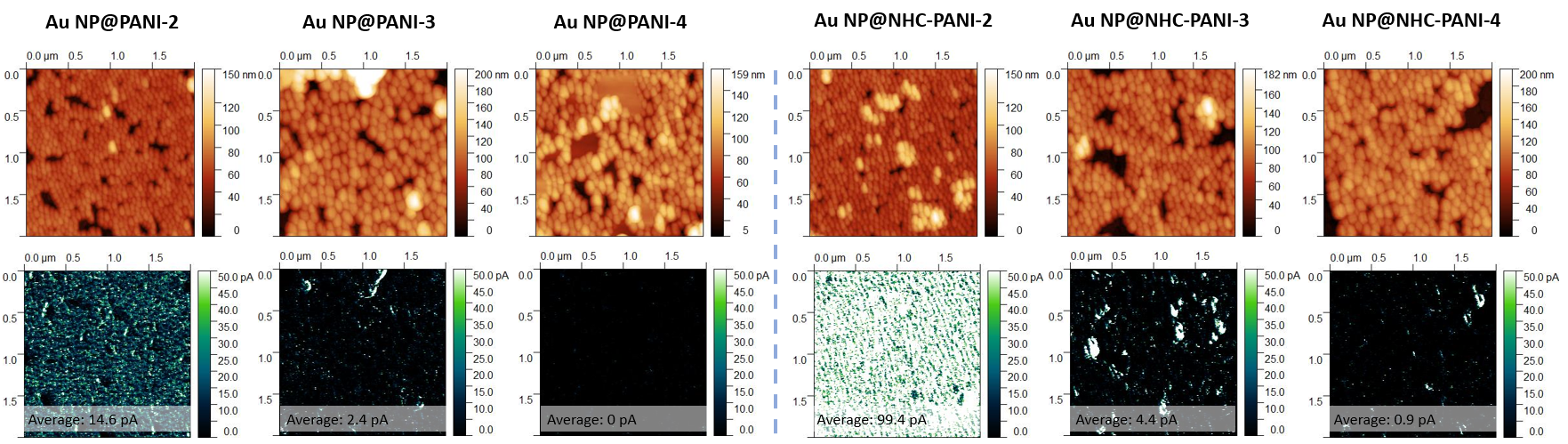


**Figure S17**. AFM (top) and C-AFM current mapping (bottom) images of Au NP@PANI (left) and Au NP@NHC–PANI (right) with different PANI shell thicknesses (PANI 2-4). The average current values extracted from the C-AFM measurements are indicated in the images.


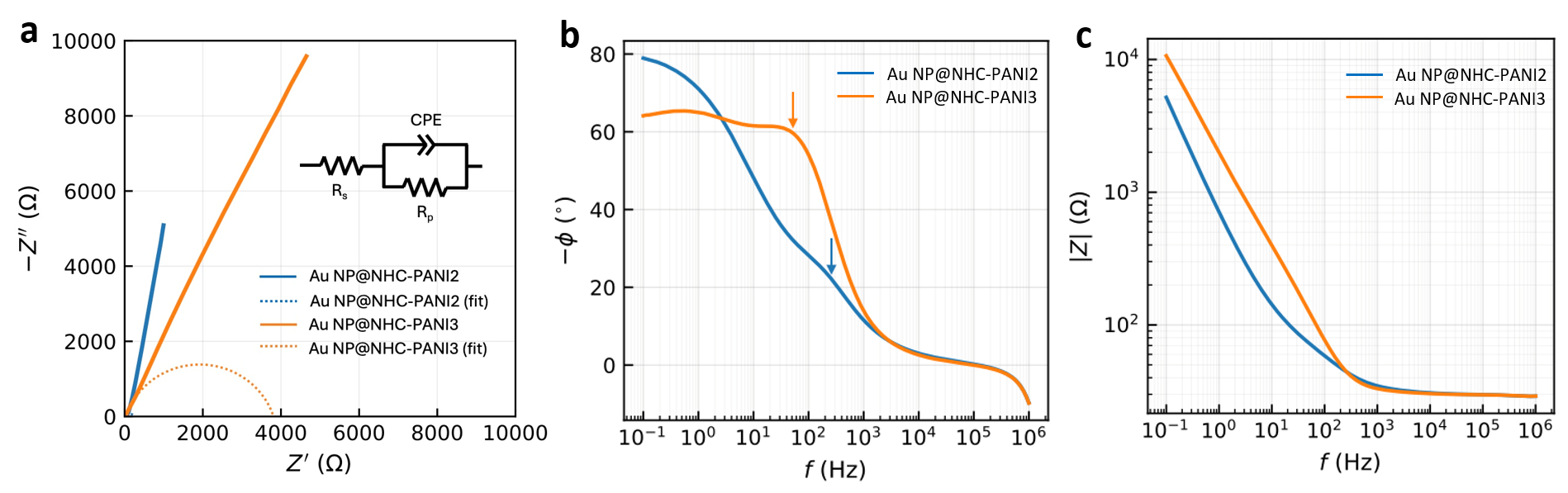


**Figure S18.** (a) Electrochemical impedance spectroscopy (EIS) characterization of the Au NP@NHC- PANI films. The Nyquist plot (solid lines) and the corresponding electrochemical circle fits (dashed lines) based on a modified Randles circuit as inserted in the plot. (b) The Bode phase plot shows the characteristic relaxation feature; the peak frequency (f_p_) is marked for each film. (c) The Bode magnitude plot illustrates the transition from high-frequency resistive behavior to low-frequency capacitive response.

**DFT calculations**

DFT calculations are used to investigate the molecular structure. For geometry optimization, we used the DFT method as implemented in the CP2K software package (cp2k.org) with the Quickstep module. ^[S6]^. All calculations were implemented under the Perdew-Burke-Ernzerhof exchange-correlation functional, ^[S7]^ the Goedecker-Teter-Hutter pseudo-potentials^[S8]^ and the valence double-ζ basis sets, in combination with the DFT-D2 method of Grimme^[S9]^ for van der Waals (vdW) correction. Molecular geometric optimization is computed without a solvation model, while the experiments are done in a dry state. We used 3 layers of gold, where the upper layer was allowed to be relaxed (planar supercell 29.8 x 19.9 Å, vacuum size 40 Å, maximum force 4.5 x 10-5 a.u.). The calculated data was analyzed, and the images were generated by the PyMOL Molecular Graphics System, Version 2.4 open-source build, Schrödinger, LLC. For DOS and transmission calculations we used the DFT+NEGF method as implemented in CP2K.


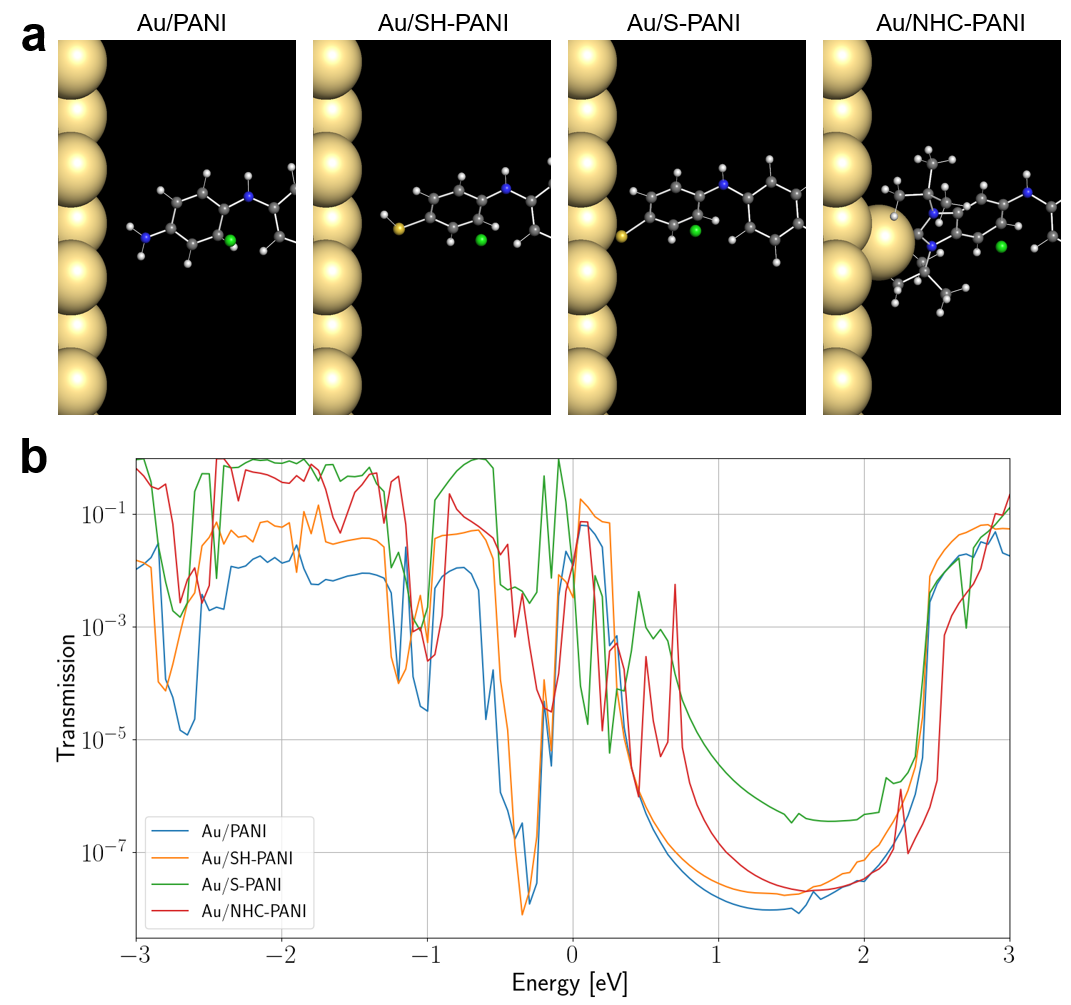


**Figure S19.** (a) Optimized junction geometries used for DFT-based transport calculations: Au/PANI (physisorbed), Au/SH–PANI (physisorbed), Au/S–PANI (chemisorbed) and Au/NHC–PANI (chemisorbed). (b) Corresponding energy-dependent transmission functions computed within the DFT+NEGF framework, comparing interfacial electronic coupling for different bonding motifs.

**
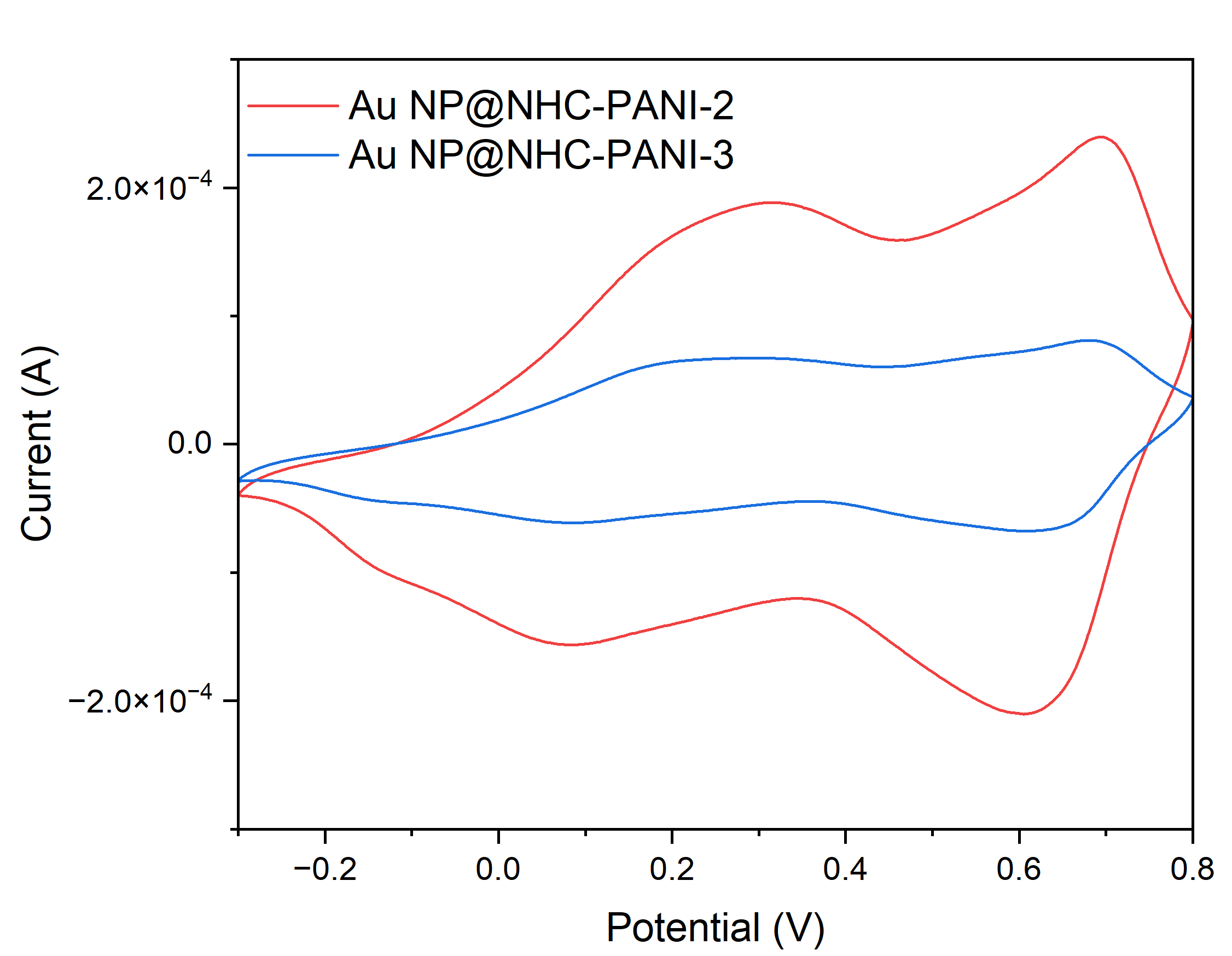
**

**Figure S20.** CV curves of Au NP@NHC-PANI-2 and Au NP@NHC-PANI-3.


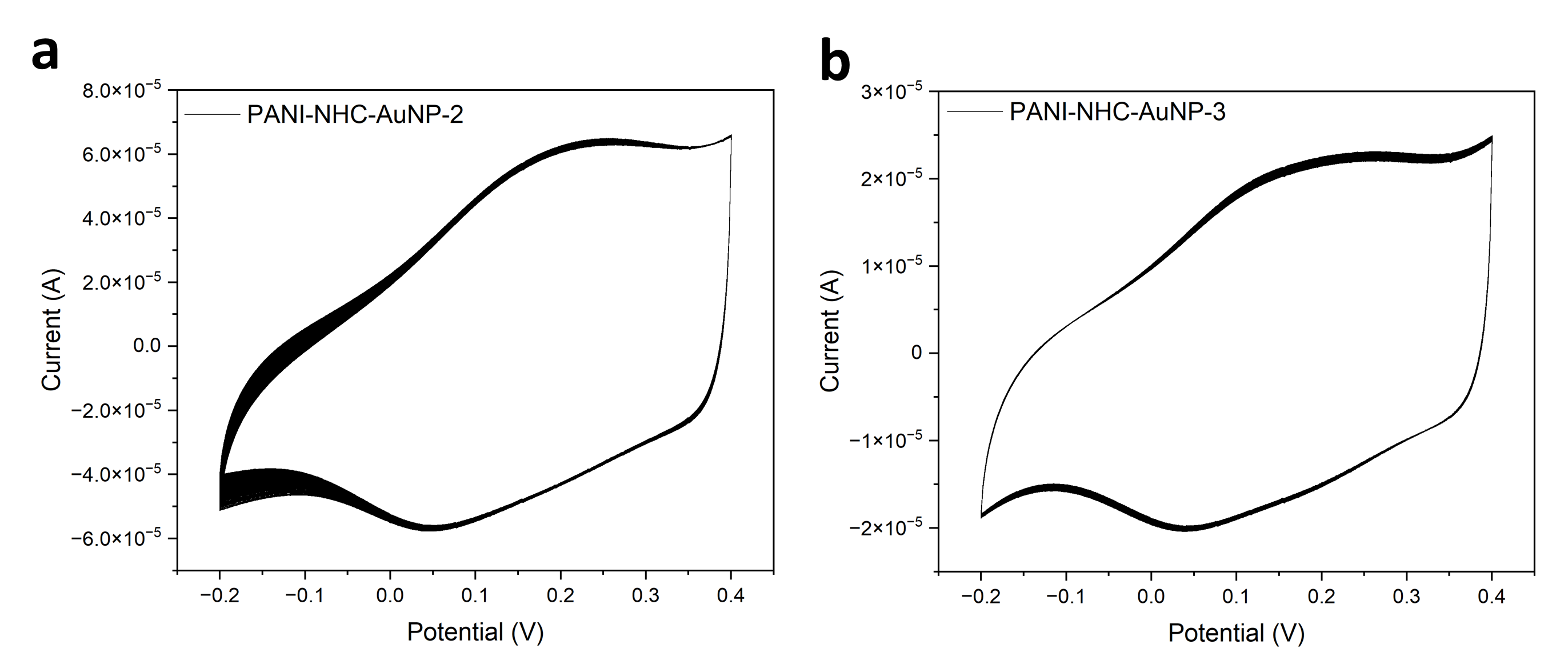


**Figure S21**. Long-term CV scans for 200 cycles of (a) Au NP@NHC-PANI-2 and (b) Au NP@NHC-PANI-3, demonstrating the electrochemical stability of the composites.


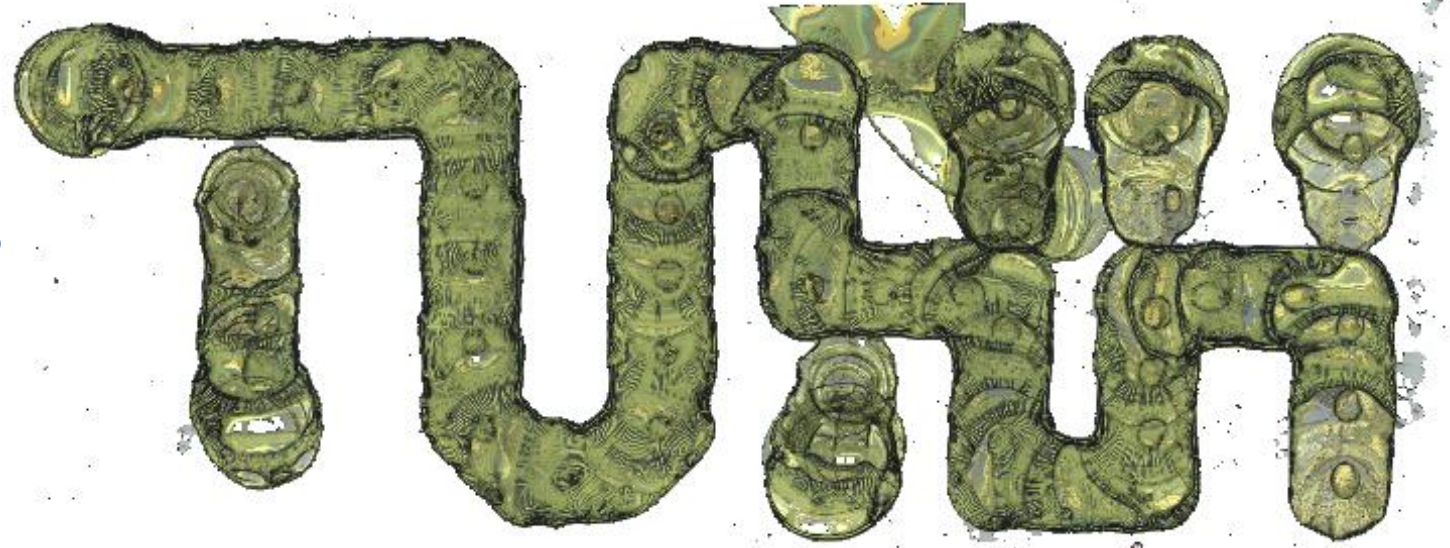


**Figure S22.** Printed TUHH lettering using doped Au NP@NHC-PANI-2 ink.


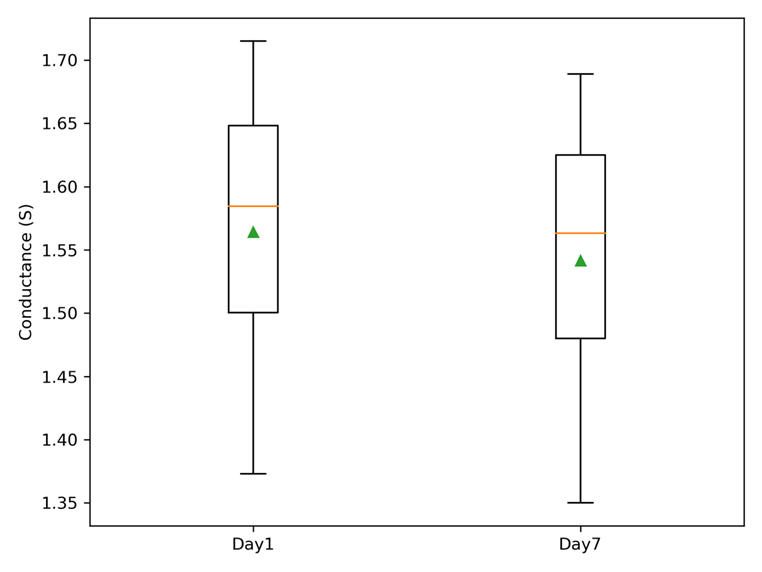


**Figure S23.** Ambient-aging stability of printed Au NP@NHC-PANI: conductance (S) measured initially and after 7 days of storage under ambient conditions.

**References**

[S1] N. G. Bastús, J. Comenge, V. Puntes, “Kinetically Controlled Seeded Growth Synthesis of Citrate-Stabilized Gold Nanoparticles of up to 200 nm: Size Focusing versus Ostwald Ripening” *Langmuir* **2011**, *27*, 11098–11105.

[S2] R. P. M. Höller, M. Dulle, S. Thomä, M. Mayer, A. M. Steiner, S. Förster, A. Fery, C. Kuttner, M. Chanana, “Protein-Assisted Assembly of Modular 3D Plasmonic Raspberry-like Core/Satellite Nanoclusters: Correl ation of Structure and Optical Properties” *ACS Nano* **2016**, *10*, 5740–5750.

[S3] J. F. DeJesus, L. M. Sherman, D. J. Yohannan, J. C. Becca, S. L. Strausser, L. F. P. Karger, L. Jensen, D. M. Jenkins, J. P. Camden, “A Benchtop Method for Appending Protic Functional Groups to N-Heterocyclic Carbene Protected Gold Nanoparticles” *Angewandte Chemie* **2020**, *132*, 7655–7660.

[S4] N. L. Dominique, P. Nalaoh, D. M. Jenkins, R. Vaia, K. Park, J. P. Camden, “One-step functionalization of gold nanorods with N-heterocyclic carbene ligands” *RSC Advances* **2025**, *15*, 5007–5010.

[S5] Patwardhan, S. V.; Emami, F. S.; Berry, R. J.; Jones, S. E.; Naik, R. R.; Deschaume, O.; Heinz, H.; Perry, C. C. Chemistry of Aqueous Silica Nanoparticle Surfaces and the Mechanism of Selective Peptide Adsorption. *J. Am. Chem. Soc.* **2012**, *134* (14), 6244–6256.

[S6] Kühne, T. D.; Iannuzzi, M.; Del Ben, M.; Rybkin, V. V.; Seewald, P.; Stein, F.; Laino, T.; Khaliullin, R. Z.; Schütt, O.; Schiffmann, F.; et al. Cp2k: An Electronic Structure and Molecular Dynamics Software Package - Quickstep: Efficient and Accurate Electronic Structure Calculations. J. Chem. Phys. 2020, 152 (19).

[S7] Perdew, J. P.; Burke, K.; Ernzerhof, M. Generalized Gradient Approximation Made Simple. Phys. Rev. Lett. 1996, 77 (18), 3865-3868.

[S8] Goedecker, S.; Teter, M.; Hutter, J. Separable Dual-Space Gaussian Pseudopotentials. Phys. Rev. B 1996, 54 (3), 1703-1710.

[S9] Grimme, S.; Antony, J.; Ehrlich, S.; Krieg, H. A Consistent and Accurate Ab Initio Parametrization of Density Functional Dispersion Correction (Dft-D) for the 94 Elements H-Pu. J. Chem. Phys. 2010, 132, 154104
